# Identity-specific reward expectations in orbitofrontal cortex guide goal-directed choices

**DOI:** 10.1371/journal.pbio.3003829

**Published:** 2026-07-09

**Authors:** Phillip P. Witkowski, Noelle Henein, Nicole Moussa, Geoffrey Schoenbaum, Thorsten Kahnt

**Affiliations:** 1 National Institute on Drug Abuse Intramural Research Program, National Institutes of Health, Baltimore, Maryland, United States of America; 2 West Virginia School of Osteopathic Medicine, Lewisburg, West Virginia, United States of America; 3 Department of Psychology, University of Maryland, College Park, Maryland, United States of America; Universitat Jaume 1, SPAIN

## Abstract

Real-life decisions are typically directed toward specific types of rewards (e.g., a slice of pizza or a bowl of pasta), but reward identity is often neglected in neuroeconomic theories of decision-making. Previous research has shown that the lateral orbitofrontal cortex (lOFC) represents the specific rewards predicted by environmental cues. However, whether and how these expectations influence decision-making remains an open question. To address these questions in humans, we developed a novel behavioral task in which Pavlovian cues associated with specific rewards are presented before participants can make decisions to forage these rewards. Using pattern-based analysis of functional magnetic resonance imaging data, we show that cues predicting distinct reward types evoke identity-specific expectations in lOFC, which in turn predict subsequent choices. This effect is amplified by activity in the nucleus accumbens, which enhances the influence of lOFC reward expectations on action representations in the dorsal anterior cingulate cortex. These results connect representational and motivational accounts of decision-making, highlighting the neural mechanism by which expectations about reward identity guide goal-directed behavior.

## Abstract 2

How reward identity representations influence choice remains unclear in neuroeconomic decision-making models. This fMRI study shows that identity-specific expectations in the human lateral orbitofrontal cortex predict choices, with nucleus accumbens amplifying their impact on action representations in dorsal anterior cingulate cortex, linking reward identity to goal-directed decision processes.

## Introduction

Behavior in the real world is almost exclusively directed toward specific types of outcomes (e.g., a slice of pizza, a trip to Norway, a neuroscience degree, etc.). The specific identity of rewards, however, is often neglected in neuroeconomic theories of decision-making, which focus solely on the abstract value of choice options. In this study, we test how the brain represents specific types of expected reward, and how these expectations guide decision-making.

The orbitofrontal cortex (OFC) plays a critical role in supporting flexible decision-making [1–5]. Evidence in both humans and other animals shows that lateral OFC encodes the identity of expected rewards [6–9]. These specific expectations could be used to bias decision-making toward rewards that are currently available in the environment [5]. Consistent with this, lesions and inactivation of lateral orbitofrontal cortex (lOFC) impair goal-directed behavior [10–14]. Moreover, work using specific Pavlovian-to-instrumental transfer (sPIT) tasks suggests that the lOFC supports goal-directed behavior in concert with the amygdala [15–18], which is thought to store memories of specific rewards [19]. Despite this evidence implicating lOFC and amygdala, we do not know whether and how identity-specific expectations in these areas guide decision-making.

One possibility is that the lOFC guides decisions by broadcasting reward identity information to downstream regions, biasing action selection towards choices that achieve these rewards. The dorsal anterior cingulate cortex (dACC) is well positioned to translate reward expectations into concrete action plans. In contrast to the lOFC, which primarily represents the identity and sensory features of expected rewards [2,5,20], the dACC signals the relative value of behavioral options [21–25]. Through dense reciprocal connections with the OFC [26–28], the dACC may serve as a computational hub that turns reward expectations into action values [29].

Such interactions between the lOFC and dACC may be further facilitated by brain areas involved in motivation. A strong candidate is the nucleus accumbens (NAc), which is thought to potentiate reward-guided responses [30,31]. NAc neurons respond to reward-predictive cues [32] and dopaminergic pathways within the NAc promote reward-guided actions for specific rewards [33–37]. However, how NAc interfaces with areas coding reward expectations and actions is unknown.

We designed a novel task to test how identity-specific reward expectations influence subsequent decision-making. We show that reward-predictive cues bias decisions towards specific reward identities and that the strength of reward identity expectations in lOFC predicts subsequent choices. Further, we show that NAc activity moderates the relationship between lOFC reward expectations and action representations in the dACC. Together, these results bridge representational and motivational accounts of decision-making, by characterizing neural mechanisms through which identity-specific reward expectations drive goal-directed behavior.

## Results

### Experimental design and behavioral task

**Fig 1:**
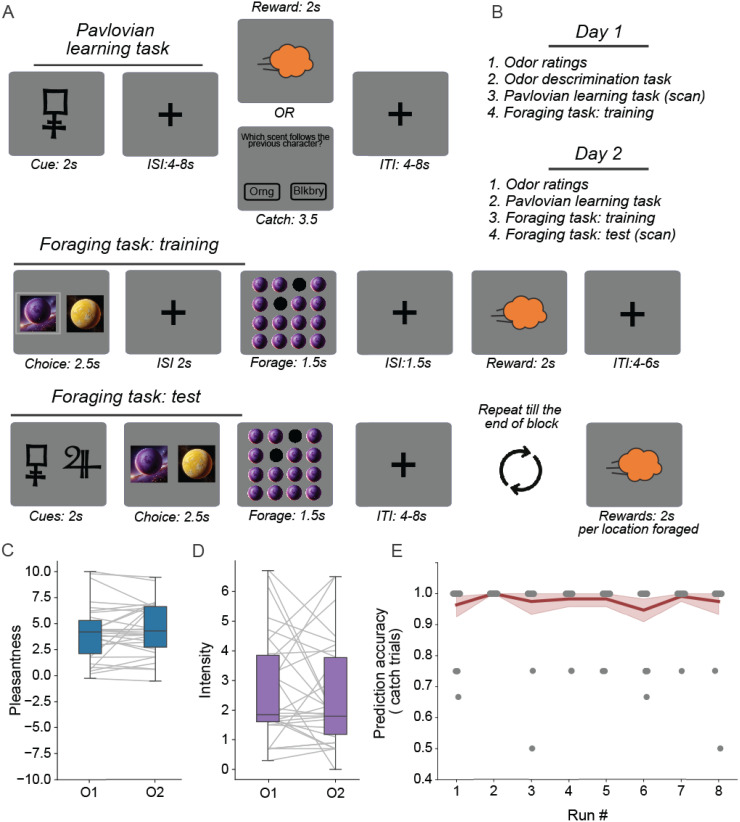
Experimental design and task. **A)** Schematic of the Pavlovian learning task and foraging tasks. In the Pavlovian learning task, participants learned to associate four cues with two pleasant food odors by passively viewing cue-to-odor pairs. In catch trials, we probed these associations by asking participants to predict what odor would come after a given cue. In the training portion of the foraging task, participants chose between two planets then foraged as many times as possible in 1.5 s. Each location on the foraging screen had a 60% chance of returning a reward. As long as at least one reward was foraged, the participants were given the odor associated with the planet at the end of the trial. The test portion of the foraging task was the same, except that participants were presented with two cues before selecting a planet to forage. These cues could either be “aligned” (predicting the same outcome) or “competing” (predicting different outcomes). Further, participants did not receive any rewards until the end of the block. DALL·E was used to create the planet stimuli. **B)** Order of tasks in each of the two days. Session days could be up to two weeks apart. **C)** Post-selection pleasantness ratings (from −10 = least pleasurable sensation imaginable, through 0 = neutral, 10 = most pleasant sensation imaginable) for the two reward odors for each participant on Day 1. **D)** Same as C but for intensity ratings (0 = very weak to 10 = extremely strong). Source data for C and D can be found at https://osf.io/24dkw/files/76uzn. **E)** Accuracy of responses for probe trials in the Pavlovian learning task. Red line represents average accuracy across participants and shading is 95% CI.

Thirty hungry participants (fasted at least 4 hours) completed the experiment. The behavioral task (Fig 1) consisted of three phases (Pavlovian learning, foraging-training, and foraging-test), which were spread across two days, no more than two weeks apart. Day 1 began with participants completing the Pavlovian learning task inside the scanner. In this task, participants learned to associate abstract visual cues with one of two desirable food odors via Pavlovian conditioning (Fig 1A). A unique pair of food odors (e.g., orange and popcorn) was chosen for each participant such that odors were discriminable and approximately equally desirable according to participants’ pleasantness ratings (Fig 1C and 1D). Two cues predicted one odor (O1), and two cues predicted the other odor (O2). The cues, as well as the designation of each odor as O1 or O2, were randomly determined at the start of the experiment. Participants learned the associations by observing each cue followed by a specific odor. We probed the formation of cue-reward associations by asking participants to predict which odor would be delivered after a cue in catch trials interspersed throughout the task (see Methods; Fig 1A). Odor predictions on catch trials during the Pavlovian learning task showed that participants quickly learned the associations between cues and odors, reaching 100% prediction accuracy by run two and maintained high accuracy for the rest of the task (Fig 1E). All runs had prediction accuracies significantly above chance (all *p* < 0.001).

After the Pavlovian learning task, participants left the scanner and completed the training phase of a foraging task. On each trial, participants could choose between two planets (presented on the left and right side of the screen) on which to forage for rewards by pressing a left or right button. There were 6 planets in total, and on three of them O1 was available, and on the other three, O2 was available. On each trial, the two planets presented on the screen (left and right position randomized) were associated with different odor identities. Once a planet was selected, participants foraged as many locations on the planet as possible to find a reward that was presented at the end of the trial. This design mimics traditional animal versions of the task in which animals must choose and then press a lever to achieve a specific reward [15,38]. Participants’ decisions during free-choice trials were biased toward planets with more pleasant odors on Day 1 (*r* = 0.32, *t*(29)=1.77, *p* = 0.044). This demonstrates that the planet-to-odors mappings were well learned, and that this knowledge was used to make value-based decisions in this task (S1A Fig).

On Day 2, participants completed shorter versions of Pavlovian learning task and the training phase of the foraging task with similar behavioral results (S1C and S1D Fig). After this, participants moved to the scanner where they completed the test phase of the foraging task. Here, we made key changes to traditional sPIT tasks which allowed us to test how reward expectations drive decision-making. First, each trial began with two of the Pavlovian cues being presented bilaterally on the screen before the planets were shown, which temporally separated the onset of cue-induced expectations from the decisions. Participants were not informed beforehand about the presentation of the two cues. Second, trials were separated into two conditions. On “aligned” trials, both Pavlovian cues predicted the same odor identity. On “competing” trials, the two cues predicted different odor identities, allowing for competition between cues. Together, these conditions allowed us to localize identity-specific reward expectations in the brain and to determine whether the strength of these expectations biased decision-making during choice.

### Identity-specific Pavlovian cues bias goal-directed choices

**Fig 2:**
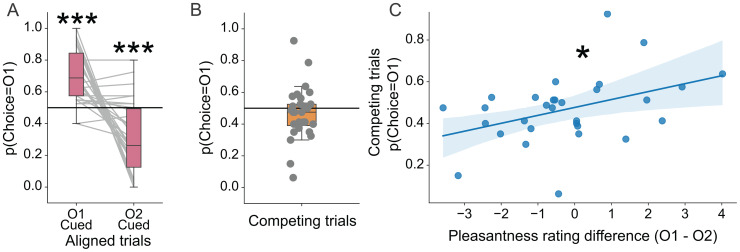
Identity-specific Pavlovian cues bias goal-directed choices. **A)** Participant choices for planets associated with odor 1 when cued for O1 or O2 in the aligned condition. Black line represents unbiased (i.e., chance) responding. Source data can be found at https://osf.io/24dkw/files/pse58. **B)** Participant choices for O1 in the competing condition. Black line represents unbiased responding. Source data can be found at https://osf.io/24dkw/files/g2vsr. **C)** Participant choices for O1 in the competing condition are correlated with the difference in pleasantness ratings between O1 and O2 from ratings on Day 2. * indicates *p* < 0.05; *** indicates *p* < 0.001.

Our central prediction was that identity-specific reward expectations evoked by the Pavlovian cues in the ‘aligned’ condition would bias decision-making towards rewards that matched those predicted by the cue. To test this, we computed the frequency of choices for planets associated with the same odor as the previously shown Pavlovian cues. Participants were more likely than chance (i.e., 50%) to select planets associated with O1 when O1 cues were presented (*t*(29)=6.79, *p* = 9.23 × 10^−8^; Fig 2A), and were biased away from O1 (towards O2) when O2 cues were presented (*t*(29)=−4.63, *p* = 3.52 × 10^−5^), consistent with previous findings [15,16,38]. The strength of this overall bias effect was not related to pleasantness or intensity differences between the odors (both *p* > 0.53, both BF_01_ > 17.98). This result demonstrates that the Pavlovian cues induced choice biases based on the specific identity of the reward rather than their hedonic value.

In contrast to the aligned condition where both Pavlovian cues predicted the same reward, the competing condition was designed to create a situation where the two Pavlovian cues would evoke two opposing reward expectations that would compete to bias goal-directed choices. On a given trial, choices in this condition should be driven by the stronger of the two reward expectations. Because the strength of reward expectations could fluctuate from trial-to-trial, we anticipated a choice distribution around chance (i.e., 50%). Indeed, the pattern of choices in this condition was consistent with this prediction overall (O1 chosen mean = 46.7%, *t*(29) = −1.09, *p* = 0.28). Moreover, the majority of participants showed only slight deviations from 50% in this condition (Fig 2B), and the strength of this deviation was correlated with the difference in pleasantness ratings for O1 over O2 (*r* = 0.38, *t*(27) = 2.11, *p* = 0.023, Fig 2C), but not with intensity ratings or the interaction between pleasantness and intensity (both ps > 0.35).

### Lateral OFC represents the identity of expected outcomes prior to decision-making

Previous work has implicated the lOFC and amygdala in goal-directed behavior [12,15,18,39]. However, it is not clear what information is represented in these regions *prior* to the onset of goal-directed choices. Based on previous studies [6,7,17,40,41], we predicted that cue-evoked activity in lOFC and amygdala would represent expectations about the identity of the reward associated with Pavlovian cues.

**Fig 3:**
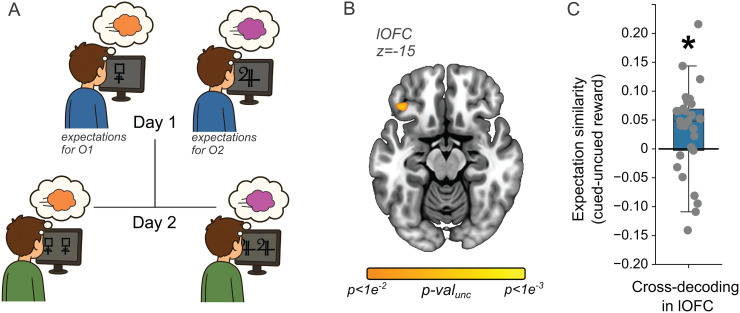
Lateral OFC represents the identity of expected reward prior to decision-making. **A)** Schematic of the analysis decoding identity-specific reward expectations at the time of the Pavlovian cue. We created a template by averaging activity patterns evoked by cues in the Pavlovian learning task for each reward identity. We then used these patterns to decode reward expectations evoked by cues in the aligned condition during the test phase of the foraging task. This graphic was created using DALL·E assistance. **B)** Axial slice of a t-statistic map showing pattern similarity for cued minus uncued reward expectations prior to choice in the test phase of the foraging task. For illustration, map is displayed at a threshold of threshold at *t*[29] = 2.462, *p* < 0.01, uncorrected. **C)** The results of the cross-decoding analysis controlling for cue-identity in an ROI constructed from significant voxels in B. We followed the same procedure but restricted our analysis to decoding trials that did not share the same cues as the template trials. Black line represents chance decoding. Source data can be found at https://osf.io/24dkw/files/5mb8v * indicates *p* < 0.05.

To test this, we constructed two identity-specific “expectation templates” by computing the multivoxel patterns of fMRI activity evoked by cues predicting each of the two rewards during the Pavlovian learning task (Day 1, Fig 3A). These templates averaged across specific cues, thus capturing purely Pavlovian reward expectations (O1 versus O2) before participants had learned any choice–reward contingencies in the foraging task. We then correlated these two expectation templates with the activity patterns evoked by the same Pavlovian cues in the test phase of the foraging task (Day 2). The trial-wise “expectation similarity” was computed as the difference between these correlation coefficients (cued minus uncued [i.e., not presented] reward identity). Positive correlation values indicated higher pattern similarity with the cued reward identity, while negative values indicated higher pattern similarity with the uncued reward identity.

We implemented this analysis in a searchlight approach within ROIs of the lOFC and amygdala, focusing on trials in the aligned condition where choices were biased towards rewards predicted by the Pavlovian cues. This analysis revealed significant decoding of identity-specific reward expectations in the left lOFC ([*x*,*y*,*z*] = [−40,31,−18], *t*[29] = 3.12, pTFCE = 0.033; Fig 3B). These decoding results could not be explained by absolute differences in pleasantness or intensity between the two expected rewards (both pTFCE > 0.5, both BF_01_ > 21; S2A and S2C Fig). We also confirmed that this effect was not driven by visual information about the cues by adopting a cross-decoding approach within an ROI consisting of significant voxels from the previous analysis (threshold at *t*[29] = 2.462, *p* < 0.01). In this analysis, expectation templates were also constructed from cue-evoked response patterns from the Pavlovian learning task, but for each of the four cues independently. We then correlated the template patterns with activity patterns evoked in the foraging task, but only for trials that did not contain the relevant cue. This showed significant decoding of identity-specific reward expectations (*t*[29] = 2.32, *p* = 0.014, Fig 3C), which was similarly not driven by differences in pleasantness or intensity ratings (both *p* > 0.25, both BF_01_ > 22.00, S2B and S2D Fig). We did not find significant decoding of identity-specific reward expectations in the amygdala (pTFCE > 0.5). An exploratory whole-brain analysis identified an effect in visual cortex ([*x*,*y*,*z*] = [−15,−87,−8], *t*[29] = 6.46 pTFCE < 0.001), which was no longer significant when controlling for visual information (*t*[29] = 0.438, *p* = 0.332).

### Identity-specific reward expectations in lOFC bias subsequent choices

The previous results show that lOFC represents identity-specific reward expectations prior to choice. A critical piece of our hypothesis is that these outcome expectations bias goal-directed choices towards rewards with the same identity. To test this hypothesis, we analyzed patterns of fMRI activity arising from the presentation of Pavlovian cues in the competing condition, in which the cues may compete for influence over behavior. If identity-specific reward expectations in lOFC drive goal-directed choices, expectation-related activity patterns corresponding to O1 should be stronger than those corresponding to O2 prior to the selection of O1 planets, and vice versa for O2.

**Fig 4:**
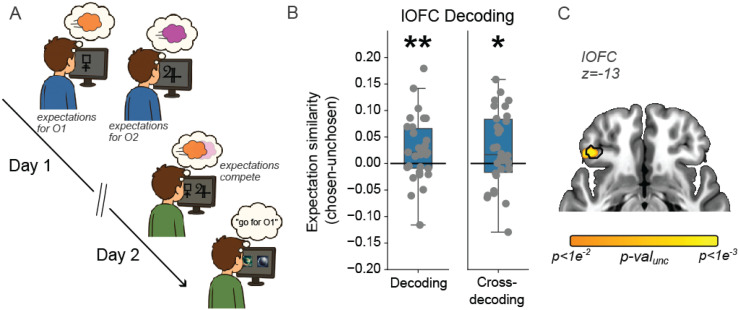
Identity-specific reward expectations in lOFC bias subsequent goal-directed choices. **A)** Schematic of the analysis shown in B and C. We compared identity-specific template pattern from the Pavlovian learning task (cf. Fig 2A) to patterns evoked by competing cues during the test phase of the foraging task, predicting that stronger decoding of one pattern over the other would correspond to subsequent goal-directed choices for that reward at the time of decision. This graphic was created using DALL·E assistance. **B)** Decoding results in lOFC ROI in Fig 2B. Left, decoding based on template constructed from all Pavlovian cues. Right, decoding when the templates and the test cues did not share visual information. Source data can be found at (right) https://osf.io/24dkw/files/q8ept; (left) https://osf.io/24dkw/files/7kpd8. C) Axial slice of t-statistic map showing the overlap of voxels that significantly predict choice from identity-specific reward expectations in the competing condition (warm colors) and voxels encoding identity-specific reward expectations in the aligned condition (black outline, from Fig 2B). ** Indicates *p* < 0.01, * Indicates *p* < 0.05.

We used the same decoding approach as described above, correlating the cue-evoked activity pattern from each competing trial in the foraging task with the expectation template patterns corresponding to O1 and O2 from the Pavlovian learning task. We then separated trials based on the choice participants would subsequently make at the time of decision (“go for O1” versus “go for O2”, Fig 4A). Expectation similarity for the chosen reward was measured as the difference in the correlation coefficients between the chosen and unchosen outcome (i.e., O1 minus O2 in “go for O1” trials, and O2 minus O1 in “go for O2” trials). Positive values indicated that the activity patterns in lOFC more strongly represented the reward identity that the participant ultimately went on to select on that trial. In other words, this analysis allowed us to test whether trial-by-trial variability in lOFC activity patterns predicted which reward participants subsequently pursued, effectively linking pre-choice identity-specific reward expectations to subsequent decisions.

We focused our analysis on voxels in the lOFC which had shown identity-specific reward expectations in the previous analysis of the aligned condition. This analysis showed that activity patterns in our lOFC ROI during presentation of the Pavlovian cues significantly predicted the reward identity that participants went on to select at the time of choice (*t*[29] = 2.51, *p* = 0.009; Fig 4B). This effect persisted when controlling for visual information via cross-decoding (*t*[29] = 2.21, *p* = 0.018). However, this analysis was somewhat sensitive to ROI definition. Using a leave-one-participant-out cross-validation procedure for defining this ROI showed only a marginally significant effect for decoding (*t*[29] = 1.656, *p* = 0.054), but a significant effect when cross-decoding (*t*[29] = 1.841, *p* = 0.038), indicating that the strength of these effects may depend on the ROI definition (see S3 Fig).

Implementing this analysis in a searchlight revealed a significant cluster in our lOFC ROI ([*x*,*y*,*z*] = [−45,31,−15], *t*[29] = 3.90, pTFCE = 0.015; Fig 4C), showing that even a cluster-agnostic analysis resulted in a highly significant overlap between voxels coding identity-specific expectations and those predicting choice (DICE coefficient = 0.559, *p* < 0.001 permutation test). We did not find any significant positive correlations between any of these decoding results and differences in rated odor pleasantness or intensity (all p’s > 0.12, all BF_01_ > 2.14, see S2E–S2H Fig). Further, we did not find any significant decoding at the whole-brain level (pTFCE > 0.588). Together, these results directly connect identity-specific reward expectations in lOFC at the time of the cue to decision biases at the time of choice.

### NAc activity relates identity-specific expectations to choices

Previous work has shown that the NAc responds to reward-predicting cues and is necessary for goal-pursuit in the presence of such cues. Here, we sought to extend this work by asking whether NAc activity is related to the degree to which (a) Pavlovian cues, and (b) the identity-specific reward expectations these cues evoke in lOFC, bias choice behavior. For this, we took a between-participants approach, as participants who show a strong cue-induced choice bias, by definition, have a large asymmetry in trials where they did versus did not choose the same reward as the cue, rendering such comparisons within-participants inappropriate.

**Fig 5:**
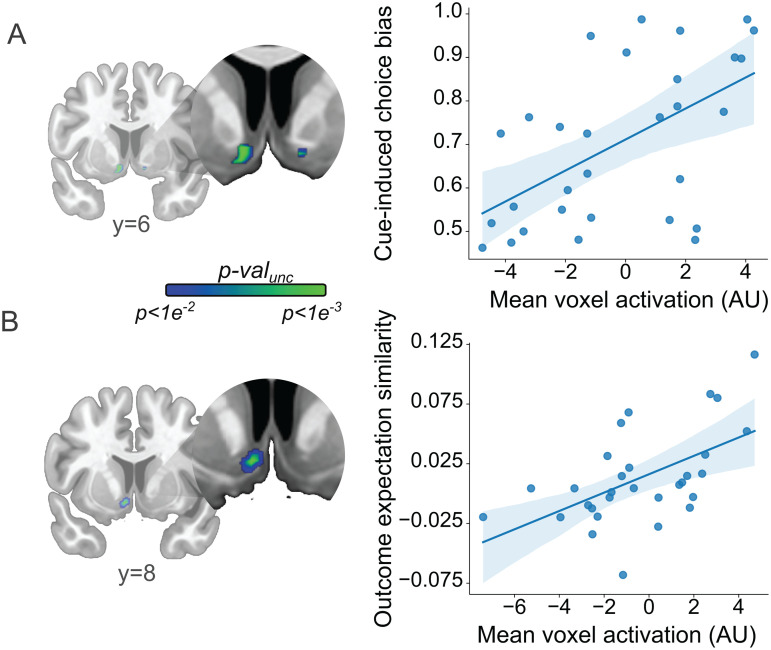
NAc activity relates identity-specific expectations to choices. **A)** Left shows a coronal slice from a t-statistic map thresholded using the same conventions as Fig 3 (peak voxel is significant at pTFCE = 0.009). Right is an illustration of the relationship between the strength of the cue-induced choice bias in the aligned condition and mean activity of the NAc at cue onset. Each dot is one participant. **B**) Left shows a coronal slice from a *t*-statistic map thresholded using the same conventions as Fig 3 (peak voxel is significant at pTFCE = 0.015). Right shows an illustration of the relationship between the predictability of choice from lOFC reward expectation patterns and mean NAc activity at cue onset. Each dot is one participant.

To test the first question, we correlated NAc BOLD responses to cues in the aligned condition with the cue-induced choice bias across participants (see Univariate Model in Methods). To control for potential confounds related to value, we also included the absolute difference in pleasantness between rewards as a covariate. Paralleling previous work [36,37], we found a significant positive correlation between cue-induced choice bias and activity in NAc at cue onset within an *a priori* ROI of the NAc ([*x*,*y*,*z*] = [−10,6,−8], *t*[29] = 4.18, pTFCE = 0.008; Fig 5A).

Next, we sought to specifically link these NAc responses to the degree to which choices in the foraging task could be predicted from the strength of identity-specific reward expectations in the lOFC. We correlated NAc activity in the aligned and competing conditions with the overall alignment between lOFC expectation patterns and subsequent choices (Fig 5B). We also included the absolute difference in pleasantness ratings as a covariate in this analysis. We observed a significant effect in the same NAc ROI ([*x*,*y*,*z*] = [−7,8, −10], *t*[29] = 3.62, pTFCE = 0.018), showing that participants with greater NAc activation during cue presentation also exhibited greater predictability of choices from lOFC expectation patterns. This correlation did not differ between the aligned and competing conditions (Fisher *z*-test *p* = 0.569), suggesting that neither condition independently drove the effect. Finally, we ran exploratory whole-brain versions of both analyses but did not find any significant clusters (all pTFCE > 0.184). Together, these results suggest that NAc activity mediates the biasing effect of Pavlovian cues on choices, potentially by gating or amplifying the influence of reward-specific expectations in the lOFC on subsequent goal-directed decision-making [35,36].

### NAc relates outcome expectations in lOFC to action representations in dACC

Thus far our results show that the lOFC encodes identity-specific reward expectations, that these expectations bias subsequent choices, and that this bias is moderated by NAc activity. These findings suggest that NAc activity may relate reward expectations in the lOFC to the selection of actions at the time of choice. This further implies that the relationship between identity-specific reward expectations in lOFC and the strength of chosen action representations should be modulated by NAc activity.

We tested this idea by first identifying brain regions that encoded the action (left versus right button press) executed by participants in each trial of the foraging task. Based on prior work showing that dACC encodes the value of specific actions [23,29], we predicted that actions would be decodable in this area. We used a searchlight approach at the time of choice and leave-one-run-out cross-validation to create “action” template patterns from all but one run (see Methods). We then used these templates to decode left versus right actions in the left-out run, by correlating trial-wise patterns with each template. We iterated this procedure over all runs and then took the average correlation across runs for each searchlight. The results showed significant action decoding in our *a priori* defined dACC ROI ([*x*,*y*,*z*] = [11,41,20], *t*[29] = 3.77, pTFCE = 0.017).

In an exploratory analysis, we also ran this searchlight across all voxels in the frontal lobe and basal ganglia (see Regions of Interest in Methods) and found significant effects in left motor cortex ([*x*,*y*,*z*] = [−45,−17,55], *t*[29] = 5.93, pTFCE = 0.001) and anterior ventromedial prefrontal cortex (vmPFC, [*x*,*y*,*z*] = [13,61,−10], *t*[29] = 4.85, pTFCE = 0.040). Follow-up analysis in each region using a finite impulse response GLM for decoding identity-specific reward expectations and actions from individual fMRI time points within a trial confirmed that reward expectations in lOFC preceded action representations in all regions (S4A Fig).

**Fig 6:**
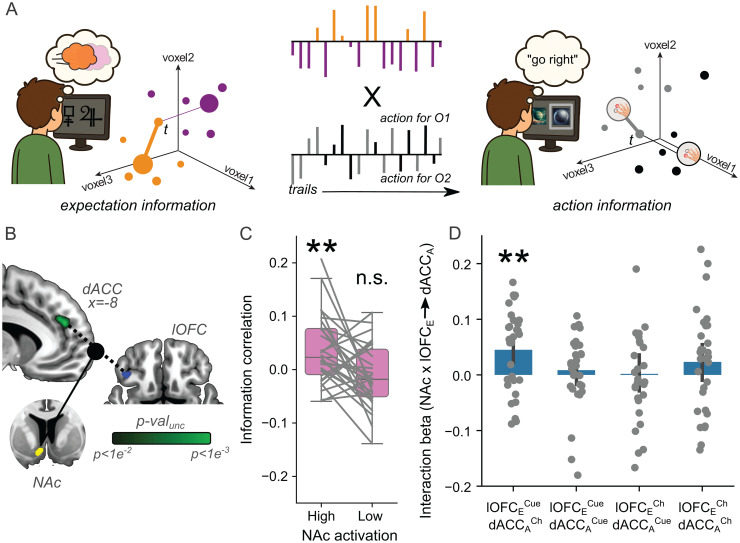
NAc relates reward expectations in lOFC to action representations in dACC. **A)** Schematic of the trial-by-trial information measurement of identity-specific reward expectations (left) and action (right). In each trial, we computed the relative distance of neural activity patterns to each of two “expectation” templates. Closer distance to one template (orange line) was taken to be an indicator of stronger expectations for that associated reward. We did the same for action representations at the time of choice (black = left, gray = right) but used a leave-one-out procedure to construct “action” templates by which the representations could be compared. These distances were then projected into the reward identity space such that they reflected the amount of information about the action required to pursue a specific reward (see Methods). This graphic was created using DALL·E assistance. **B)** Schematic and *t*-statistic maps showing the results of the information connectivity analysis conducted on the information metrics computed in A. We used a trial-by-trial regression framework to test whether the relationship between lOFC expectation strength and dACC action representations depended on NAc activity, effectively testing NAc’s moderating influence on information transfer between lOFC reward expectation and dACC action representations. Maps are displayed using the same conventions as Fig 3. **C)** Illustration of results shown in C, separated by median-split based on NAc activity, showing that lOFC outcome expectations are positively correlated with dACC action information on trials with high NAc activity. Source data can be found at https://osf.io/24dkw/files/kh4uq. **D)** Estimated interaction coefficients for alternative temporal orderings of information connectivity. lOFC_E_ represents the trial-by-trial time course of decoded reward expectations in lOFC and dACC_A_ represents the time course of decoding actions in dACC. Superscripts indicate the time point at which these representations were decoded, “Cue” is at the time of the cue and “Ch” is at the time of choice. Source data can be found at https://osf.io/24dkw/files/jpvzc ** indicates *p* < 0.01.

To test whether identity-specific reward expectations drive representations of the chosen action during choice, we regressed the strength of decoded reward expectations in the lOFC at the time of the Pavlovian cue onto chosen action representations in the dACC at the time of choice. First, we derived values for the relative strength of identity-specific reward expectations in lOFC on a trial-by-trial basis by computing the pattern similarity between the lOFC activity pattern on each trial to each expectation template. The difference between these similarity values was taken to be the relative strength of the expectation (Fig 6A). Stronger O1 expectation patterns had positively signed values, while stronger O2 expectation patterns had negatively signed values. Again, we focused our analysis on lOFC voxels that represented identity-specific reward expectations in the aligned cue condition.

We then calculated the strength of action representations at the time of choice by comparing activity patterns on each trial to the mean activity pattern for left and right responses, respectively. The difference between these similarity values was taken to be the strength of the action representation on each trial. However, we changed the sign of the decoded action representation to be positive if that action selected a planet where O1 could be obtained, and negative for planets where O2 could be obtained. Thus, a positive relationship between the time series of expectation strength and action strength representations would mean that strong identity-specific reward expectations in the lOFC are associated with strong dACC representations of actions that obtain the same specific outcome.

Based on our previous findings that NAc activation at the time of the cue was related to the magnitude of cue-induced choice biases (Fig 5), we included trial-by-trial fluctuations in cue-evoked NAc activity as a moderator on this association. Finally, our regression model also included a regressor accounting for the identity of the reward pursued on each trial (see Methods).

We found a significant positive interaction between lOFC expectations and NAc activity in predicting the strength of action representations in dACC ([*x*,*y*,*z*] = [−15,46,20], *t*[29] = 3.43, pTFCE = 0.041, Fig 6B). We obtained the same result when restricting the analysis to voxels that significantly represented chosen action ([*x*,*y*,*z*] = [10,13,38], *t*[29] = 2.96, pTFCE = 0.028). Follow up analysis showed that this effect did not differ between aligned and competing trials (pTFCE > 0.33). No other effects were found in a whole-brain analysis (pTFCE > 0.105). To illustrate and confirm this effect, we plotted the correlation between lOFC expectations and dACC action representations separately for trials with high and low NAc activity (median split Fig 6C). This shows that this interaction effect was driven by a significant positive correlation between lOFC outcome expectations and dACC action representations when NAc activity was high (*t*[29] = 3.05, *p* = 0.005), and no significant correlation when NAC activity was low (*t*[29] = −1.24, *p* = 0.22). This effect was specific to the dACC and was not found in motor cortex or anterior vmPFC (both pTFCE > 0.1). Further, the effect in these voxels was specific to the relationship between lOFC expectations at the time of cue and dACC action representations at the time of choice and was not significant for alternative temporal orderings (all ps > 0.08, all BF_01_ > 38.95; Fig 6D). Similar effects were also not significant when using activity in other subcortical areas instead of NAc (see S1 Table). These results suggest that NAc activity may amplify the influence of Pavlovian cues on choice by strengthening the link between identity-specific reward expectations in lOFC and action representations in dACC.

## Discussion

Our results provide novel insights into the neural mechanisms that support decision-making for specific rewards. We show that reward expectations in the lOFC bias subsequent choices and delineate the pathway through which they influence actions. Specifically, we show that NAc facilitates cue-induced choice biases and promotes the transfer of information between the lOFC and dACC. Together, these results inform neuroeconomic theories of decision-making, offering evidence for the functional role of reward expectations in lOFC.

Our central hypothesis was that lOFC represents specific reward expectations prior to choice [2,5,6] and that these representations would govern decision biases at the time of choice. Consistent with this hypothesis, we found that Pavlovian cues not only evoke specific reward expectations, but that the strength of these expectations predict which reward participants will pursue at the time of choice. Further, our results show that multiple cues may compete for influence in the lOFC, with the stronger expectation determining which reward is subsequently pursued. This finding fundamentally extends prior work in non-human primates showing that OFC representations of option value are only weakly related to subsequent choices [42], and highlights how value-orthogonal information can bias decisions. Note that we did not find representations of identity-specific outcome expectations in an exploratory whole-brain analysis, suggesting that these effects may be restricted to the lOFC. These results place the lOFC at the center of an active decision-making process in which the identity of specific options is a critical decision variable.

Importantly, follow-up analyses revealed additional information about the anatomical localization of these effects within lOFC. The cluster of voxels showing the choice-prediction effect survived correction within an anatomical ROI of lOFC, but the effect was not significant across the entire ROI, suggesting it is restricted to a subregion within lOFC. This cluster significantly overlapped with voxels coding identity-specific expectations, but the overlap was moderate, and constraining the choice-prediction analysis to voxels that represented identity-specific expectations was somewhat sensitive to the exact selection criteria (S3 Fig). Together, this suggests that areas of lOFC coding identity-specific reward expectations when cues predict the same reward may only partially overlap with those recruited to resolve competition when multiple cues signal different reward expectations.

The role of lateral OFC in signaling specific rewards contrasts with adjacent medial OFC and medial prefrontal cortex, which represent relative value signals that are independent of the identity of choice options [43–45]. These functional differences are likely supported by different patterns of anatomical connectivity, as lOFC shares stronger connections with sensory areas compared to mPFC [27]. These functional and anatomical differences point to distinct, but complementary, roles in decision-making processes which rely on similar coding mechanisms. Specifically, the mPFC codes subjective value in a relative format, where increases in activity correspond to higher chosen value relative to the unchosen option. Our results show a corresponding coding scheme for reward identity in the lOFC where the strength of multivariate representations of the chosen reward identity was stronger compared to the unchosen reward identity. These parallels point to a potentially conserved read-out process for decision variables even with disparate coding schemes, where stronger signals bias decisions to the corresponding option.

In contrast to previous findings in rodents [15,18] and humans [16,46], we did not find evidence that the amygdala is involved in the effect of Pavlovian cues on choice behavior. However, prior work in humans focused on linking amygdala BOLD responses to single cue biases [16], consistent with models of amygdala function which propose a role in linking specific reward memories to specific cues during learning [47] and using these memories for decision-making [19,41]. Thus, it could be that the presentation of two cues in each trial made it difficult to detect cue-unique patterns of activity, whereas lOFC has been shown to integrate over cues to form expectations [48]. Future work is needed to parse the potential differences and similarities between predicted reward coding in these regions.

Importantly, our results elaborate how a network of regions, including the NAc and dACC, contribute to transforming lOFC representations into choice biases and ultimately action selection. Specifically, we show that NAc activity promotes cue-induced choice biases and facilitates the propagation of information from reward expectations in lOFC to action representations in the dACC, linking studies that have shown contributions of these regions in isolation [9,36,37,49,50]. This stands in contrast to an exploratory analysis suggested by one of the reviewers, which did not reveal evidence for a moderating effect of amygdala, mediodorsal thalamus, or perirhinal cortex on the information connectivity between lOFC and dACC. These findings highlight the role of the NAc, a motivational center heavily innervated by dopaminergic projections [51–53] and innovator of motor regions [35,54], in facilitating the influence of identity-specific reward expectations on action selection signals. Together, our findings connect research which has often focused on the necessity of individual regions by showing that the lOFC, dACC, and NAc work together to transform environmental information into goal-direct behaviors.

Our behavioral results build on a history of behavioral neuroscience research utilizing tasks with specific outcomes, most notably specific Pavlovian-to-instrumental transfer [15,55,56]. The current task design advances this work in two fundamental ways. Participants in our task were presented with Pavlovian cues before the opportunity to make choices, paralleling real-world situations where predictive cues and choices are often temporally separated (e.g., freeway billboards and the exit to the restaurant). This advances previous work by showing that the influence of cues is not limited to immediate choices but extends at least several seconds into the future. Second, our task dissociates decisions from specific actions, requiring “on the fly” mapping between specific goals and actions. This shows that cue-evoked outcome expectations can be flexibly directed to actions that achieve these outcomes, and do not depend on learned action-outcome mappings. Both of these advances suggest that previous findings from sPIT tasks may be generalizable to more realistic situations in which cues (e.g., advertisements) bias behavior even when they are no longer present and when achieving these goals requires the flexible use of different actions.

In summary, our findings offer a mechanistic account of how cues predicting specific rewards influence decision-making. Identity-specific reward representations in lOFC are related to biases in goal-pursuit decisions. These biases are moderated by activity in the NAc and flexibly mapped onto reward-obtaining action signals in the dACC. These findings call for neuroeconomic theories of decision-making that emphasize the role of reward identity—not only their abstract value—in shaping adaptive behavior.

## Methods

### Ethics statement

The study was approved by the National Institute of Health Institutional Review Board (Study Nr. 001156) and written informed consent was obtained from all participants. All procedures in this study were conducted according to the principles expressed in the Declaration of Helsinki.

### Participants

Thirty participants (21 Female; mean age = 28 y/o, sd = 5.55 years) were recruited from Baltimore, MD, and surrounding areas. Participants fasted for at least 4 hours before (mean fasting time = 12 hours 3 min, sd = 7 hours 37 min) the study and received monetary compensation for participation.

### Odor stimuli

Twelve food odors were used during the study (pot roast, potato chip, sautéed onion, orange, apple, passion fruit, blackberry, popcorn, brownie, pineapple, cinnamon roll, cheesecake). The odors were delivered to participants using a custom-built computer-controlled olfactometer capable of redirecting medical-grade air with precise timing at a constant flow rate of 2.97 L/min through the headspace of glass bottles containing liquid solutions of the food odors. The olfactometer was equipped with two independent mass flow controllers (Alicat, Tucson, AZ), allowing for the dilution of odorants with odorless air. There was a constant stream of odorless air delivered to participants’ noses through a nasal mask throughout the experiment. The odors were mixed into this airstream at specific time points, without any change in the overall flow rate. Consequently, odor presentation did not involve a change in somatosensory stimulation.

### Odor selection and discrimination task

For each participant, we found a bespoke pair of two odors to be used as rewards for the experiment. The odors were determined by using a “odor rating task”, in which participants rated the pleasantness of food odors using a hedonic scale [57], which ranges from “least pleasurable sensation imaginable” to “most pleasant sensation imaginable”. In between these anchors were non-linearly spaced verbal descriptors of ratings, but participants could place the rating marker anywhere in between. Each trial began with the presentation of a fixation cross, followed by a 3-s countdown which preempted the release of the odor. At the end of the countdown, the central cross turned blue, indicating to participants to sniff the odor. After the blue cross disappeared, the rating scale appeared, and participants indicated the pleasantness of the odor. Participants were given unlimited time to make their selection (median RT = 4.92s). Each odor was presented and rated four times. Two final odors were selected such that the odors were as positively rated as possible while the difference in their ratings was as minimal as possible.

To ensure that participants were able to discriminate the selected odors, they were asked to complete a discrimination test. Each trial was made up of two odor presentations punctuated by an 8-s delay interval. The odors within each trial were either the same odor presented twice or two different odors. Each odor presentation was preceded by a 3-s countdown and the cross turned blue simultaneously with the onset of the odor. Participants indicated whether the odors were different or the same by clicking buttons on the screen that read “same” or “different”. This was done four times each for three conditions (“outcome 1 – same”, “outcome 2 – same”, “different odors”). If a participant scored equal to or less than chance (50%) in any one condition, new odors were selected, and the process was repeated until two discriminable odors were found (Overall accuracy = 97.7%). Odors which passed these criteria were used as rewards for the rest of the experiment.

### Pavlovian learning task

Participants learned associations between cues and odors during an initial learning session. Cues were a set of four characters pre-selected from the Agathadomian character set. Each trial began with a cue displayed for two seconds at the center of the screen, followed by an interstimulus interval (ISI) which lasted from 4 to 8 s uniformly distributed. At the end of the ISI, the central fixation cross turned from black to blue, indicating the odor had been released. Participants had 2 s to sniff the odor and were explicitly instructed their goal was to learn “which odor came after each cue”. Two cues were paired with O1 and the other two paired with O2. Trials ended with a 4–8 s jittered intertrial interval, uniformly distributed.

We intermittently tested participants knowledge of the association in “catch trials”. These trials followed the same sequence as learning trials, except that rather than an odor being delivered after the ISI, participants were shown buttons with the odor names on either side of the screen and asked to indicate which odor comes after the previously shown cue. The odors names appeared on each side of the screen in random order. Responses were indicated by pressing the left or right buttons, corresponding to each side of the screen.

Each cue-odor combination was presented four times in each run. Catch trials appeared only at the end of the run for the first two runs. For the rest of the runs, catch trials were randomly mixed with learning trials, and participants were not informed about when they would be presented.

### Foraging task—Training phase

After the Pavlovian learning task, participants completed a “foraging game”, in which they played as interstellar travelers looking for food for their long journey. Participants learned the types of “food” that could be foraged on 6 different planets by choosing between 2 planets on each trial then foraging in as many “locations” as possible. The six planets were randomly organized into three pairs of planets consisting of one planet which led to O1 and the other led to O2. Each trial began with one pair of planet images presented on the screen with a randomly determined side. Participants were given 2 s to choose which planet to travel by pressing either the right or left arrow key with their right hand. Their selection stayed on the screen for another 500 ms plus any remaining time in the response period.

After a 1-s ISI, a five-by-five gird of small planet icons appeared on the screen, representing different locations to forage. Participants were instructed to forage as many locations as possible by pressing with their middle finger on their left hand as many times as possible in 1.5 s. Each time the key was pressed, a random location was considered ‘foraged’ and turned black. Each location had a 60% chance of producing a rewarding odor. For the purposes of training, we only presented one odor at the end of the trial if at least one odor had been found. At the end of the foraging period, the icons remained on the screen for an additional second. After another 2-s ISI, the odor associated with the planet was presented concurrently with a blue fixation cross. Trials ended with a central black fixation cross displayed for 4–6 s (uniform distribution), before the start of the next trial.

To ensure that participants learned about all planets, we included forced choice trials at the beginning of each block. Here, a white box appeared around one of the planets indicating which planet should be chosen. Participants were still required to make a response, but only responses for the highlighted planets were accepted. All planets had an equal number of forced-choice and free-choice presentations, and trials where participants did not respond within the response window were repeated at the end of the block.

### Foraging task—Test phase

This task was the same as the foraging training task except that each trial began with the presentation of two Pavlovian cues horizontally distributed on the screen. These cue pairs could indicate either the same (aligned condition) or different rewards (competing condition). The number of aligned trials consisting of the same cue twice versus different cues was also equivalent. Participants were not given any instructions about these cues and were not told they would appear before the task. Trial types were counter-balanced, such there were an equivalent number of trials with aligned conditions and competing conditions. The number of aligned cue pairs that consisted of the same cue twice versus two different cues were also equivalent. The cues were presented for 2 s before an ISI, which consisted of presenting a fixation cross for 2 s.

After the ISI, the trials proceeded in the same fashion as during the training phase, with one critical exception: no outcome was presented after the foraging phase. This was to prevent any observed effects being confounded by new learning. Participants were made aware that they would no longer receive odors at the end of each trial. Instead, participants were instructed that at the end of each run, one trial would be randomly selected and all rewards they won on that trial would be delivered (i.e., they could receive multiple deliveries of an odor). Thus, their goal was to accumulate as many rewards as possible in every trial to maximize the possibility of a high number of reward deliveries. Each trial ended with a 4- to 8-s ITI where only a fixation cross was presented. Participants did not repeat trials in which they failed to respond during planet selection (missed trials Mean = 1.5%, SD = 1.6%). Once the run was completed, participants received all outcomes from foraging (i.e., both rewards and non-rewards) from a single randomly selected trial. If no rewards were accumulated during foraging, then participants received only clean air.

### Procedure

The experiment was broken into two sessions that occurred on separate days. Participants were instructed not to eat 4 hours prior to each session and reported doing so (time since last meal days 1 and 2: mean = 12 hours SD = 7.5 hours). Day 1 began with the odor selection and discrimination tasks outside of the scanner. After the reward odors were chosen, participants rated each odor for pleasantness and intensity. Participants then completed 8 runs of the Pavlovian learning task inside the scanner, followed by 6 runs of the training phase of the foraging task outside the scanner.

Day 2 began with participants rating the pleasantness and intensity of the odors before completing “reminder” trials of the Pavlovian learning task and training phase of the foraging-training task. Reminder trials for each task were the same as the previous day, except there were only 4 blocks of Pavlovian learning trials and 4 blocks of foraging task trials. Both occurred outside of the scanner. After this, they completed 10 runs of the test phase of the foraging task in the scanner. Both occurred outside of the scanner. Day 1 and Day 2 were separated by a minimum of 1 day and a maximum 14 days (mean = 2 days, SD = 2.02 days).

### fMRI data acquisition

MRI data were acquired on a Siemens 3T PRISMA system equipped with a 32-channel head-coil. Echo-Planar Imaging (EPI) volumes were acquired with a parallel imaging sequence with the following parameters: repetition time, 1.5 s; echo time, 12 ms, 29 ms, 45 ms, and 62 ms; flip angle, 67°; multi-band acceleration factor, 4; slice thickness, 2.5 mm; no gap; number of slices, 60; interleaved slice acquisition order; matrix size, 104 × 96 voxels; field of view 208 mm × 192 mm. The functional scanning window was tilted ~30° from axial to minimize susceptibility artifacts in OFC. Each run of the Pavlovian learning task consisted of 202 EPI volumes, while each run of the foraging task consisted of 179 EPI volumes. A 1 mm isotropic T1-weighted structural scan was also acquired for each participant and used for spatial normalization.

### fMRI data preprocessing

All image preprocessing and general linear modeling was done using SPM12 software (https://www.fil.ion.ucl.ac.uk/spm/). To correct for motion, we first aligned all EPI images from the 18 fMRI runs to the first acquired image for images from the first echo. We then applied this realignment mapping to the three remaining echoes. We then combined images across echoes using the tSNR-weighted PAID method [58]. We then calculated the mean image across all 18 runs of the experiment, which was then co-registered to the T1 structural scan. The T1 volume was spatially normalized to the Montreal Neurological Institute (MNI) template using the 6-tissue probability map provided by SPM12. The deformation fields resulting from this normalization were applied to the co-registered task run EPI’s, bringing these into alignment with the standard MNI coordinate system. For the univariate analysis, images were smoothed with a 6 × 6 × 6 mm FWHM Gaussian kernel, and for multivariate data images were smoothed with a 2.5 × 2.5 × 2.5 mm FWHM Gaussian kernel.

### First-level GLM for multivariate analysis

First-level generalized linear models (GLM) were conducted according to the single-trial LS methods described by [59]. For the Pavlovian learning task, we created a unique GLM for each trial in each run with a single regressor for the cue presentation, then two more regressors which captured all other cue presentations and all other odor presentations during the run. Similarly, for the test phase of the foraging task we created a unique GLM for each time point of interest in each trial (cue period and choice phase), with a single regressor for that time point and 3 more regressors for all other cue presentations, environment selection periods, and foraging periods, respectively.

We included in each model nuisance regressors controlling for six directions of framewise displacement and rotation, their squared values, their derivatives, and the square of their derivatives (24 total). Dummy regressors indicating volumes with a framewise displacement 5 standard deviations above the mean were all added to the model to control for signals that may confound estimation of single-trial maps. We included additional control regressors for nasal air flow and nasal breath volume down-sampled to scanner temporal resolution (66 Hz). Due to a technical error, we did not record breathing data for a single run of the Foraging task—test phase in 2 participants.

### Decoding of identity-specific reward expectations

To detect identity-specific reward expectations, we began by constructing multivoxel template patterns from estimates (*t*-statistic maps) of fMRI activity taken from the first-level GLMs of the Pavlovian learning task. For each voxel, we averaged the activity across all cue presentations separately for cues representing O1 and those representing O2. All trials in run 2–8 were used in the construction of these templates to minimize noise in the pattern. We then compared single-trial pattern estimates taken from the cue period of aligned trials of the foraging task to the two template patterns using Pearson correlation distance. This resulted in a 2-by-trial number neural representational dissimilarity matrix (RDM) which was compared to a binary test RDM of the same size. In each cell of the test matrix, the value was zero if the cue presented in the currently considered trial predicted the same outcome as template, and a 1 otherwise. The neural RDM was compared to the test RDM using a Pearson correlation, which was fisher *z*-transformed prior to second-level testing.

We used this method to conduct a searchlight procedure for patterns in the lOFC and amygdala containing information about the expected outcome. We used an 8 mm radius spherical searchlight across all voxels within *a priori* lOFC and amydgala ROIs (see Regions of Interest in Methods). Each participant’s map was smoothed with 6 × 6 × 6 mm Gaussian Kernal and submitted to a one-sample *t* test for group-level comparisons. We corrected for multiple comparisons using threshold-free cluster enhancement (TFCE) [60].

We then used a cross-decoding procedure to isolate patterns with information about the reward identity independent of cue identity. We created a ROI from the voxels that coded reward expectations in the searchlight analysis (thresholded at *t*(29) = 2.462, *p* < 0.01 uncorrected). We then constructed template patterns for these voxels in the same way as described above, except that the template patterns were constructed for each individual cue. Similarly, data from the foraging task were subset such that we only included trials where identical cues were shown during the cue period. We then correlated the pattern of activity from foraging task trials with two template patterns that did not include the currently presented cue. These pattern dissimilarities were compared to a test RDM created the same way as described above. We repeated this until all combinations of relevant template patterns and foraging task activity patterns were tested and then averaged over the result. This was done for each participant, and the results were submitted to a one-sample *t* test for second-level testing.

To test whether reward expectations predicted choices in the competing condition, we again used an ROI constructed from voxels found to be significant in the initial searchlight analysis, but compared them to competing trials from the test phase of the foraging task. Here, the test RDM was constructed by labeling each trial by which outcome (O1 or O2) the participant would go on to select at the time of choice. We did these using templates constructed by averaging over cues associated with the same reward and separately using the cross-decoding procedure described above. The resulting Pearson correlations were then submitted to one-sample *t* test after being Fisher *z*-transformed. A complementary searchlight analysis was also performed with the same searchlight procedure as we did for the aligned trials within the same *a priori* defined lOFC ROI.

### Decoding action representations

We used a similar decoding procedure to decode action representations during the test phase of the foraging task in frontal cortex and the basal ganglia. Here, we used a leave-one-run-out scheme to construct templates and test them on a held-out set of trials. We began by making template representations for each action by averaging all single-trial activity patterns separately for left and right button press from all runs except one. Each trial in the left-out run was then compared to each of the resulting templates using Pearson correlation distance. This created a 2×N neural-RDM which was compared to a binary test RDM with zeros in cells where the trial action matched the template action and 1 where it did not. The neural RDM and test RDM were compared using a Pearson correlation.

We used a searchlight procedure to detect voxels in the brain that represent actions at the time of choice. The searchlight was an 8 mm radius sphere around a central voxel. Correlation coefficients for all voxels were Fisher-*z* transformed before being submitted to a one-sample *t* test for second-level testing. We corrected for multiple comparisons using TFCE in an independently defined dACC ROI, and in a mask of frontal and basal ganglia regions for exploratory analysis (see Regions of Interest).

### Information connectivity analysis

To test whether information about identity-specific reward expectations were related to action representations in prefrontal cortex, we calculated correlations in trial-by-trial fluctuations in representational strength between each type of information [39,61]. To obtain a measure of the strength of reward expectations, we focused on the relative strength of the identity specific reward expectation representations elicited by cues during the cue period of test phase of the foraging task. We conducted the analysis in an ROI generated from voxels that had previously shown significant decoding of reward expectations (thresholded at *t*(29) = 2.462, *p* < 0.01 uncorrected). We constructed expectation template patterns by taking the mean pattern of activity elicited by cues associated with the same outcome over all trials in blocks 2–8 of the Pavlovian learning task. We then compared the pattern of activity in each trial of the foraging task to each of the templates using correlation distance. The difference in the distance to each template was taken as the relative information about a particular reward expectation.

Similarly, fluctuations in the representational strength of actions were computed as the relative distance to two action template representations during the test phase of the foraging task. Templates were constructed using a leave-one-run-out scheme and tested on a held-out set of trials. We began by making template representations for each action by averaging all single-trial activity patterns separately for left and right button presses from all runs except one. Each trial of the left-out run was then compared to each of the resulting templates using correlation distance, and the difference between distance to each template was taken as the relative strength of each action representation. Importantly, these distances were then signed according to the reward this action *would have achieved* if it had been taken. This means that there will be a positive correlation between regions that show strong action representations for actions which achieve rewards indicated by identity-specific expectation representations in lOFC, and a negative if there are stronger representations of the other action. We calculated distance using a searchlight approach with 8 mm radius searchlight across regions of the brain that had previously shown strong decoding of action information (*t*(29) = 2.462, *p* < .01).

Finally, we computed trial-by-trial fluctuations of NAc activity by taking the mean activity over voxels that were positively related to participants pursuing rewards that matched evoked expectations in lOFC (*t*(29) = 2.462, *p* < 0.01).

We tested the association between the strength of identity-specific reward expectations and action representations using a regression model. This model predicted trial-by-trial fluctuations in the strength of action representation in each voxel from four predictor variables: trial-by-trial fluctuations of reward expectations in the lOFC, trial-by-trial fluctuations in mean activity of NAc voxels, the interaction between these two variables, and identity of the reward pursued in each trial. The latter most variable was added to show that any association between identity-specific reward expectations and action representations went above and beyond the association between lOFC expectations and choice. All variables were *z*-scored prior to being submitted to the regression model and correlation values were Fisher-*z* transformed before being submitted to a one-sample *t* test for second-level testing.

### Univariate analysis

We measured univariate BOLD responses to the cue by constructing participant-level event-related general linear models (GLM’s) consisting of three event-related regressors-of-interest: the cue presentation period, the choice period, and the foraging period. For the foraging period, we also included a parametric regressor that accounted for the total number button presses in each trial. Nuisance regressors included: the smoothed, normalized nasal air flow and volume which were down-sampled to scanner temporal resolution (0.66 Hz); six realignment parameters resulting from motion-correction; the derivative, square, and the square of the derivative of each realignment regressor. Additional dummy regressors were added as needed to remove the effects of volumes in which particularly strong head motion occurred. At the second level, we conducted two analyses. In the first, we regressed parameter estimates for each participant against the strength of cue-induced choice bias. In the second, we regressed parameter estimates for each participant against the strength of the association between expectation in lOFC and subsequent choices. In both cases, we also included the absolute difference between pleasantness ratings for the two odors (collected at the beginning of the second session) as a regressor.

### Regions of interest

Our *a priori* lOFC ROIs were constructed by combining clusters 4 and 5 of the *k* = 6 parcellation from Kahnt and colleagues (2012) [62], which were chosen to approximate regions that had shown significant coding of identity-specific outcome expectations in previous studies. We then identified voxels within this ROI that significantly represented identity-specific reward expectations (threshold at *t*[29] = 2.462, *p* < 0.01 uncorrected) and generated a new lOFC ROI which we used to conduct subsequent tests. For the dACC, we combined dorsal area 32 and the anterior rostral cingulate zone (indexes 5, 18, 26, 39) taken from Neubert and colleagues (2015) [63]. The amygdala ROIs were taken from Echevarria-Cooper and colleagues (2022) [64], and the NAc ROI was taken from the Anatomical Automatic Labeling (AAL) atlas (Rolls and colleagues (2020) [65]; indexes 157 and 158). For exploratory analyses, we used a mask that included all frontal and basal ganglia regions from the AAL (indexes 1–46, 75–78, 135–138, and 151–158).

## Supporting information

S1 Fig Behavioral responses during Pavlovian training and foraging task training phase. **A)** Accuracy of responses for probe trials in the Pavlovian learning task. Red line represents average accuracy across participants and shading is 95% CI. **B**) Relationship between the frequency of choosing planets associated with O1 in the foraging training task and pleasantness rating of O1 relative to O2. Shaded area is 95% CI. **C)** Same as A but for Pavlovian training of day 2. **D)** Same as B but for the foraging training task on Day 2. * indicates *p* < 0.05, *** indicates *p* < 0.001.
(PDF)

S2 Fig Control analyses for relationships between perceptual odor ratings and reward identity decoding. **A)** Relationship between decoding of reward identity expectations at the time of the cues in the aligned condition and the absolute difference in pleasantness ratings between the two rewards (*t*[29] = −0.799, *p* = 0.431, BF_01_ = 21.32). **B)** Same as A but for cross-decoding analysis (*t*[29] = 0.136, *p* = 0.893, BF_01_ = 29.60). **C)** Relationship between decoding of reward identity expectations at the time of the cues in the aligned condition and the absolute difference in intensity ratings between the two rewards (*t*[29] = −0.291, *p* = 0.773, BF_01_ = 27.17). **D)** Same C but for cross-decoding analysis (*t*[29] = −0.612, *p* = 0.545, BF_01_ = 22.35). **E)** Relationship between correlation between identity expectations and choices in the competing condition and the absolute difference in pleasantness ratings between the two rewards (*t*[29] = 0.693, *p* = 0.494, BF_01_ = 23.14. **F)** Same as E but for cross-decoding analysis (*t*[29] = 1.619, *p* = 0.117, BF_01_ = 8.97). **G)** Relationship between correlation between identity expectations and choices in the competing condition and the absolute difference in intensity ratings between the two rewards (*t*[29] = −0.903, *p* = 0.374, BF_01_ = 18.88). **H)** Same G but for cross-decoding analysis (*t*[29] = −2.49, *p* = 0.022, BF_01_ = 2.41). Results from A to H are summarized in main text. **I)** For aligned trials, we computed separate templates for O1 and O2 and then computed the correlation between trials where O1 or O2 was cued with their respective templates. For competing trials, we computed the correlation between trials where participants chose O1 or O2 and their respective templates. In each case, we then computed the difference in the correlation strength between whichever reward had the higher minus the lower pleasantness rating (i.e., High Pref. − Low Pref.). In both conditions, we found null results from independent *t*-tests (aligned *t*(29) = 1.195, *p* = 0.121, B1_01_ = 6.62; competing *t*(29) = 1.490, *p* = 0.074, B1_01_ = 4.14). **J)** Correlation between difference in decoding strength for High Pref minus Low Pref. odors and the absolute difference in pleasantness ratings for each odor (aligned *t*[29] = −0.342, *p* = 0.735, BF_01_ = 27.46; competing *t*[29] = 0.267, *p* = 0.791, BF_01_ = 27.35).
(PDF)

S3 Fig Identity-specific reward expectations in lOFC predict goal-directed choices with alternative ROI definition. We compared identity-specific template pattern from the Pavlovian learning task to patterns evoked by competing cues during the test phase of the foraging task, predicting that stronger decoding of one pattern over the other would correspond to subsequent goal-directed choices for that reward at the time of decision. Here, we define the lOFC ROI using a leave-one-out procedure. We started by finding voxels which showed significant decoding of the identity of the expected reward in the aligned condition across all but one participant (*t*[28] = 2.467, *p* < .01). We then used this ROI to compute the similarity of trials in which participants chose to pursue a specific reward with that reward’s template representation from Pavlovian training trials. We found marginally significant effect when using all Pavlovian training trials in the template (*t*[29] = 1.656, *p* = 0.054), and a significant effect when we decoded across cue-types to control for visual information (*t*[29] = 1.841, *p* = 0.038).
(PDF)

S4 Fig Time course of decoding reward expectations and actions. To determine the relative time at which expectation and action information appeared within a trial, we used a finite impulse response model to estimate the BOLD signal without convolving the HRF. Estimated signals were binned by the length of a TR (1.5 s). Templates for each decoding analysis were generated from the same expectation pattern estimates as in the main text (Fig 4). Action template patterns were generated by estimating the mean pattern of activity when pressing the right or left buttons in all trials over 9 runs, then comparing it to left-out runs. We repeated this procedure for 10 TRs after the onset of the Pavlovian cues. Colored bars at the bottom indicate TRs in which decoding corresponding to a given color is significant. All pattern comparisons were done using Pearson correlations.
(PDF)

S1 Table Alternative moderating ROIs for information connectivity analysis. To test whether other subcortical regions may be involved in information transfer from lOFC to dACC, we repeated the searchlight analysis from the main text using subcortical ROIs from the AAL (caudate, putamen, pallidum, and substantia nigra pars compacta, the amygdala, hippocampus, the medial dorsal thalamus, and the nucleus accumbens). We used the trial-by-trial univariate cue-evoked activity in each ROI as an interaction regressor with lOFC expectation decoding to predict action decoding in dACC. We found that after correction for multiple comparisons, only the NAc ROI showed a significant effect.
(PDF)

## Abbreviations

AAL: Anatomical Automatic Labeling
dACC: dorsal anterior cingulate cortex
EPI: Echo-Planar Imaging
GLM: generalized linear models
lOFC: lateral orbitofrontal cortex
ISI: interstimulus interval
MNI: Montreal Neurological Institute
NAc: nucleus accumbens
OFC: orbitofrontal cortex
RDM: representational dissimilarity matrix
sPIT: specific Pavlovian-to-instrumental transfer
TFCE: threshold-free cluster enhancement
