# Supplementary material for: Identity-specific reward expectations in orbitofrontal cortex guide goal-directed choices
Source: PLoS Biol. 2026 Jul 9;24(7):e3003829. doi: 10.1371/journal.pbio.3003829 (PMC13349123; doi:10.1371/journal.pbio.3003829)
Supplement: S1 Fig — A) Accuracy of responses for probe trials in the Pavlovian learning task. Red line represents average accuracy across participants and shading is 95% CI. B) Relationship between the frequency of choosing planets associated with O1 in the foraging training task and pleasantness rating of O1 relative to O2. Shaded area is 95% CI. C) Same as A but for Pavlovian training of day 2. D) Same as B but for the foraging training task on Day 2. * indicates p < 0.05, *** indicates p < 0.001. (PDF) [file pbio.3003829.s001.pdf]

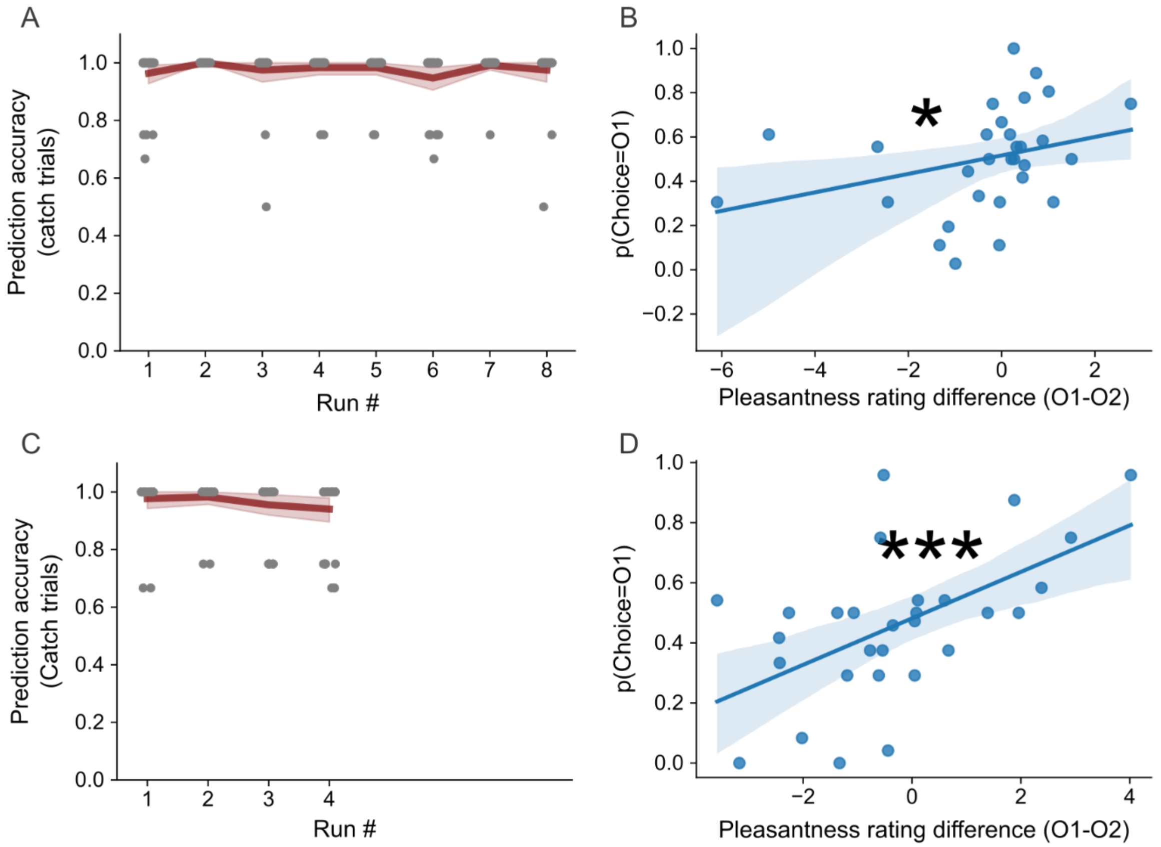

S1 Figure. Behavioral responses during Pavlovian learning task and foraging task – training phase A) Accuracy of responses for probe trials in the Pavlovian learning task. Red line represents average accuracy across participants and shading is 95% CI. **B**) Relationship between the frequency of choosing planets associated with O1 in the foraging task – training phase and pleasantness rating of O1 relative to O2. Shaded area is 95% CI. **C)** Same as A but for Pavlovian learning task of day 2 **D)** Same as B but for the foraging task - training phase on Day 2. * indicates $p<0.05$, *** indicates $p<0.001$
