# Supplementary material for: Identity-specific reward expectations in orbitofrontal cortex guide goal-directed choices
Source: PLoS Biol. 2026 Jul 9;24(7):e3003829. doi: 10.1371/journal.pbio.3003829 (PMC13349123; doi:10.1371/journal.pbio.3003829)
Supplement: S2 Fig — A) Relationship between decoding of reward identity expectations at the time of the cues in the aligned condition and the absolute difference in pleasantness ratings between the two rewards (t[29] = −0.799, p = 0.431, BF01 = 21.32). B) Same as A but for cross-decoding analysis (t[29] = 0.136, p = 0.893, BF01 = 29.60). C) Relationship between decoding of reward identity expectations at the time of the cues in the aligned condition and the absolute difference in intensity ratings between the two rewards (t[29] = −0.291, p = 0.773, BF01 = 27.17). D) Same C but for cross-decoding analysis (t[29] = −0.612, p = 0.545, BF01 = 22.35). E) Relationship between correlation between identity expectations and choices in the competing condition and the absolute difference in pleasantness ratings between the two rewards (t[29] = 0.693, p = 0.494, BF01 = 23.14. F) Same as E but for cross-decoding analysis (t[29] = 1.619, p = 0.117, BF01 = 8.97). G) Relationship between correlation between identity expectations and choices in the competing condition and the absolute difference in intensity ratings between the two rewards (t[29] = −0.903, p = 0.374, BF01 = 18.88). H) Same G but for cross-decoding analysis (t[29] = −2.49, p = 0.022, BF01 = 2.41). Results from A to H are summarized in main text. I) For aligned trials, we computed separate templates for O1 and O2 and then computed the correlation between trials where O1 or O2 was cued with their respective templates. For competing trials, we computed the correlation between trials where participants chose O1 or O2 and their respective templates. In each case, we then computed the difference in the correlation strength between whichever reward had the higher minus the lower pleasantness rating (i.e., High Pref. − Low Pref.). In both conditions, we found null results from independent t-tests (aligned t(29) = 1.195, p = 0.121, B101 = 6.62; competing t(29) = 1.490, p = 0.074, B101 = 4.14). J) Correlation between difference in decoding streng [file pbio.3003829.s002.pdf]

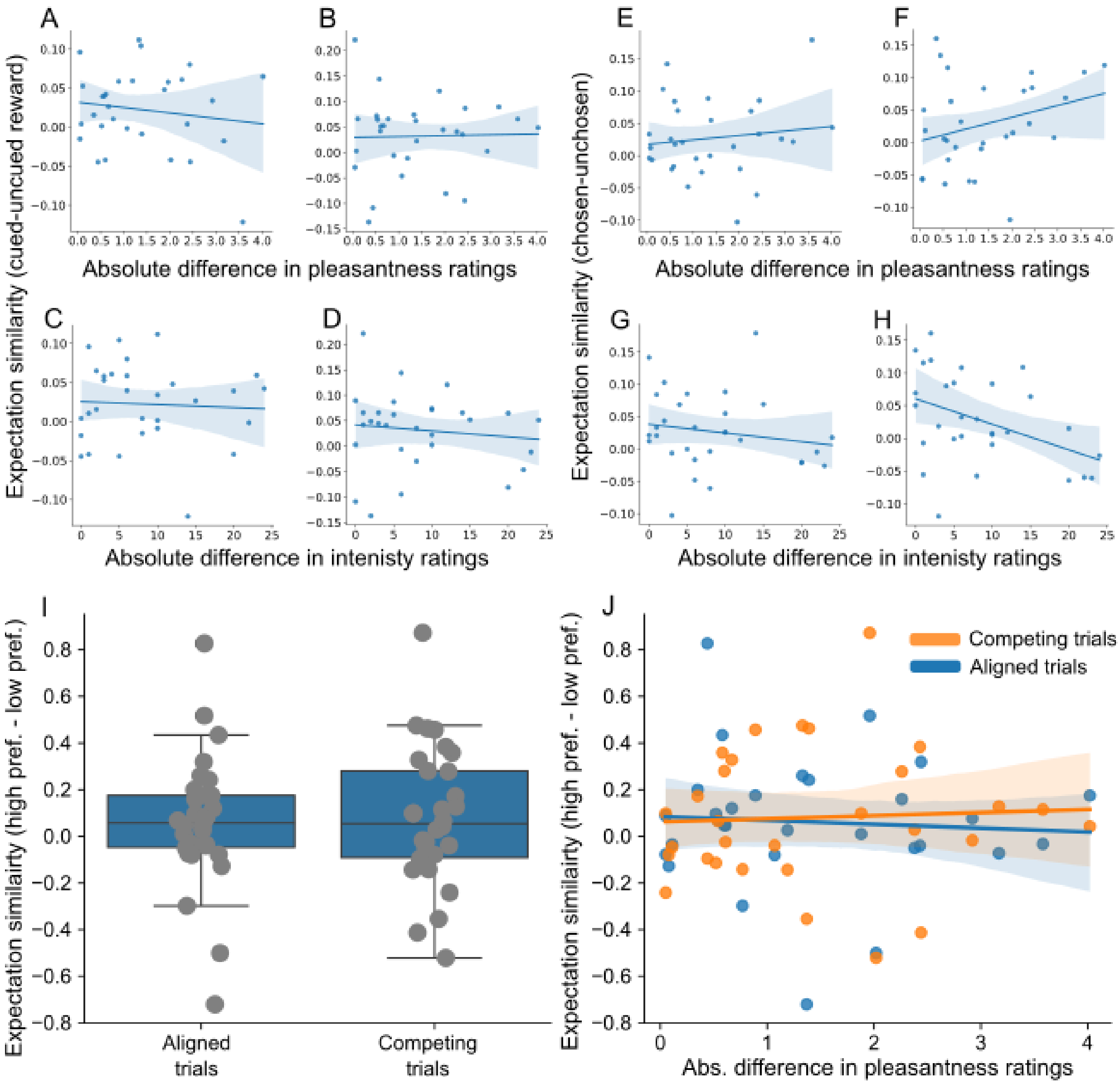

S2 Figure. Control analyses for relationships between perceptual odor ratings and reward identity decoding. A) relationship between decoding of reward identity expectations at the time of the cues in the aligned condition and the absolute difference in pleasantness ratings between the two rewards (t[29]=-0.799, p=0.431, BF_{01}=21.32). **B)** same as A but for cross-decoding analysis (t[29]=0.136, p=0.893, BF_{01}=29.60) **C)** relationship between decoding of reward identity expectations at the time of the cues in the aligned condition and the absolute difference in intensity ratings between the two rewards (t[29]=-0.291, p=0.773, BF_{01}=27.17). **D)** same C but for cross-decoding analysis (t[29]=-0.612, p=0.545, BF_{01}=22.35). **E)** relationship between correlation of identity expectations and choices in the competing condition and the absolute difference in pleasantness ratings between the two rewards (t[29]= 0.693, p=0.494, BF_{01}=23.14. **F)** same as E but for cross-decoding analysis (t[29]= 1.619, p=0.117, BF_{01}=8.97). **G)** relationship between correlation of identity expectations and choices in the competing condition and the absolute difference in intensity ratings between the two rewards (t[29]=-0.903, p=0.374, BF_{01}=18.88). **H)** same G but for cross-decoding analysis (t[29]=-2.49, p=0.022, BF_{01}=2.41). Results from A-H are summarized in main text. **I)** For aligned trials we computed separate templates for O1 and O2 and then

computed the correlation between trials where O1 or O2 was cued with their respective templates. For competing trials, we computed the correlation between trials where participants chose O1 or O2 and their respective templates. In each case, we then computed the difference in the correlation strength between whichever reward had the higher minus the lower pleasantness rating (i.e. High Pref. – Low Pref.). In both conditions we found null results from independent t-tests (aligned $t(29)=1.195$, $p=0.121$, $B1_{01}=6.62$; competing $t(29)=1.490$, $p=0.074$, $B1_{01}=4.14$). Source data can be found at osf.io/24dkw/files/syr3b **J)** correlation between difference in decoding strength for High Pref minus Low Pref. odors and the absolute difference in pleasantness ratings for each odor (aligned $t[29]=-0.342$, $p=0.735$, $BF_{01}=27.46$; competing $t[29]=0.267$, $p=0.791$, $BF_{01}=27.35$).
