# Supplementary material for: Identity-specific reward expectations in orbitofrontal cortex guide goal-directed choices
Source: PLoS Biol. 2026 Jul 9;24(7):e3003829. doi: 10.1371/journal.pbio.3003829 (PMC13349123; doi:10.1371/journal.pbio.3003829)
Supplement: S3 Fig — We compared identity-specific template pattern from the Pavlovian learning task to patterns evoked by competing cues during the test phase of the foraging task, predicting that stronger decoding of one pattern over the other would correspond to subsequent goal-directed choices for that reward at the time of decision. Here, we define the lOFC ROI using a leave-one-out procedure. We started by finding voxels which showed significant decoding of the identity of the expected reward in the aligned condition across all but one participant (t[28] = 2.467, p < .01). We then used this ROI to compute the similarity of trials in which participants chose to pursue a specific reward with that reward’s template representation from Pavlovian training trials. We found marginally significant effect when using all Pavlovian training trials in the template (t[29] = 1.656, p = 0.054), and a significant effect when we decoded across cue-types to control for visual information (t[29] = 1.841, p = 0.038). (PDF) [file pbio.3003829.s003.pdf]

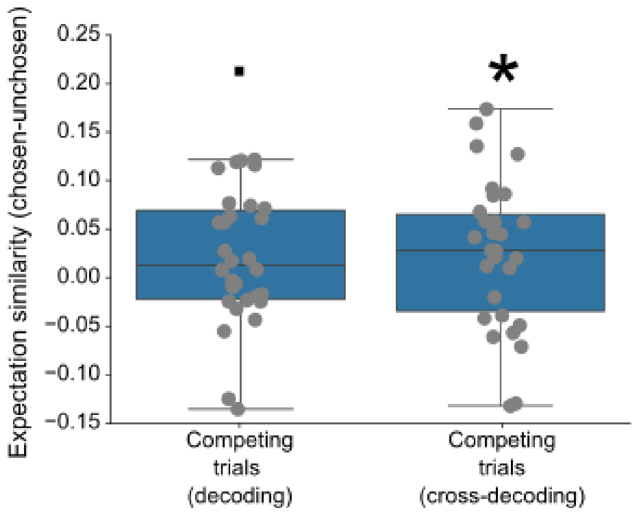

S3 Figure. Identity-specific reward expectations in lOFC predict goal-directed choices with alternative ROI definition. We compared identity-specific template pattern from the Pavlovian learning task to patterns evoked by competing cues during the test phase of the foraging task, predicting that stronger decoding of one pattern over the other would correspond to subsequent goal-directed choices for that reward at the time of decision. Here, we define the lOFC ROI using a leave-one-out procedure. We started by finding voxels which showed significant decoding of the identity of the expected reward in the aligned condition across all but one participant ($t[28]=2.467$, $p<.01$). We then used this ROI to compute the similarity of trials in which participants chose to pursue a specific reward with that reward's template representation from Pavlovian training trials. We found marginally significant effect when using all Pavlovian training trials in the template ($t[29]=1.656$, $p=0.054$), and a significant effect when we decoded across cue-types to control for visual information ($t[29]=1.841$, $p=0.038$). Source data can be found at: osf.io/24dkw/files/ncdqk
