# Supplementary material for: Identity-specific reward expectations in orbitofrontal cortex guide goal-directed choices
Source: PLoS Biol. 2026 Jul 9;24(7):e3003829. doi: 10.1371/journal.pbio.3003829 (PMC13349123; doi:10.1371/journal.pbio.3003829)
Supplement: S4 Fig — To determine the relative time at which expectation and action information appeared within a trial, we used a finite impulse response model to estimate the BOLD signal without convolving the HRF. Estimated signals were binned by the length of a TR (1.5 s). Templates for each decoding analysis were generated from the same expectation pattern estimates as in the main text (Fig 4). Action template patterns were generated by estimating the mean pattern of activity when pressing the right or left buttons in all trials over 9 runs, then comparing it to left-out runs. We repeated this procedure for 10 TRs after the onset of the Pavlovian cues. Colored bars at the bottom indicate TRs in which decoding corresponding to a given color is significant. All pattern comparisons were done using Pearson correlations. (PDF) [file pbio.3003829.s004.pdf]

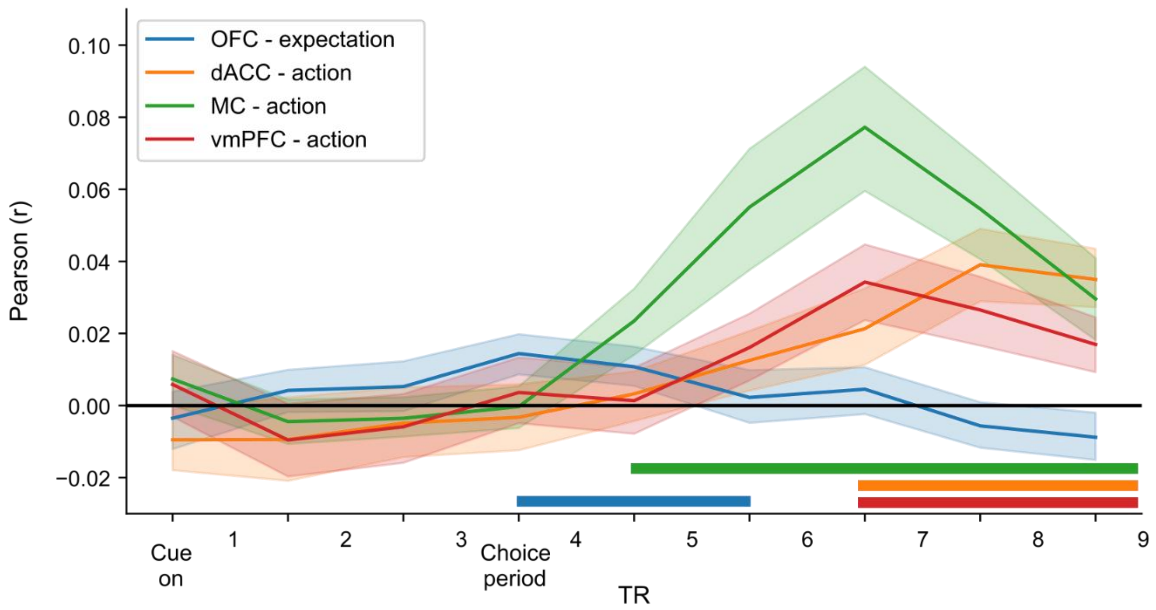

S4 Figure. Time course of decoding reward expectations and actions. To determine the relative time at which expectation and action information appeared within a trial, we used a finite impulse response model to estimate the BOLD signal without convolving the HRF. Estimated signals were binned by the length of a TR (1.5s). Templates for each decoding analysis were generated from the same expectation pattern estimates as in the main text (figure 4). Action template patterns were generated by estimating the mean pattern of activity when pressing the right or left buttons in all trials over 9 runs, then comparing it to left out runs. We repeated this procedure for 10 TRs after the onset of the Pavlovian cues. Colored bars at the bottom indicate TRs in which decoding corresponding to a given color is significant. All pattern comparisons were done using Pearson correlations.
