# Supplementary material for: Identity-specific reward expectations in orbitofrontal cortex guide goal-directed choices
Source: PLoS Biol. 2026 Jul 9;24(7):e3003829. doi: 10.1371/journal.pbio.3003829 (PMC13349123; doi:10.1371/journal.pbio.3003829)
Supplement: S1 Table — To test whether other subcortical regions may be involved in information transfer from lOFC to dACC, we repeated the searchlight analysis from the main text using subcortical ROIs from the AAL (caudate, putamen, pallidum, and substantia nigra pars compacta, the amygdala, hippocampus, the medial dorsal thalamus, and the nucleus accumbens). We used the trial-by-trial univariate cue-evoked activity in each ROI as an interaction regressor with lOFC expectation decoding to predict action decoding in dACC. We found that after correction for multiple comparisons, only the NAc ROI showed a significant effect. (PDF) [file pbio.3003829.s005.pdf]

Region	p-value	FDR
Caudate	0.2854	0.2948
Pallidum	0.2948	0.2948
Putamen	0.0468	0.1117
SN	0.0698	0.1117
Amygdala	0.0656	0.1117
MD Thalamus	0.0946	0.1261
Hippocampus	0.0134	0.0536
NAc	0.006	0.048

S1 Table. Alternative moderating ROIs for information connectivity analysis. To test whether other subcortical regions may be involved in information transfer from lOFC to dACC, we repeated the searchlight analysis from the main text using subcortical rois from the AAL (caudate, putamen, pallidum and substantia nigra pars compacta, the amygdala, hippocampus, the medial dorsal thalamus, and the nucleus accumbens). We used the trial-by-trial univariate cue-evoked activity in each ROI as an interaction regressor with lOFC expectation decoding to predict action decoding in dACC. We found that after correction for multiple comparisons, only the NAc ROI showed a significant effect.
