# Supplementary material for: Identity-specific reward expectations in orbitofrontal cortex guide goal-directed choices
Source: PLoS Biol. 2026 Jul 9;24(7):e3003829. doi: 10.1371/journal.pbio.3003829 (PMC13349123; doi:10.1371/journal.pbio.3003829)
Supplement: Attachment — Submitted filename: point-by-point responses_R1.pdf [file pbio.3003829.s008.pdf]

Reviewer #1 (MaryAnn Noonan signed her report): Witkowski and colleagues present a paper that examines how reward identity guides choice. They identify a network of regions that transfer reward identity into actions. Central to that is process is the lOFC which codes identify-specific expectations that are related to the subsequent choice. Subsequent analyses suggest these signals are amplified by the nucleus accumbens and potentially translated into action representations in the dACC.

Overall, I think this was an excellent study. The study aims are clear; the task was well designed and the data analysed appropriately. I therefore have limited comments and suggestions.

Thank you for taking the time to review our manuscript and the positive comments.

1. The description of the foraging task was a little unclear to me. In particular why did participants had to forage for the different odours? Did the participants get just a single odour regardless of how many locations they foraged? In standard PIT tasks this would be the part of the task in which the action-outcome associations are formed but here it seems like the action is not important but instead subjects learn another stimulus-outcome association. Is this how the reader should interpret this component of the task?

Thank you for this comment because it gives us a chance to clarify and highlight some aspects of the task we feel are important. We designed this task to mimic specific PIT (sPIT) designs in which animals may decide between two levers to achieve a specific reward (e.g. Sias et al. 2018), such that in our task selecting a planet was akin to selecting a lever and foraging was akin to pressing the lever to gain a specific reward. We have noted this on page 4:

> This design mimics traditional animal versions of the task in which animals must choose and then press a lever to achieve a specific reward.[15,38]

More generally, the planet selection part of the task can be interpreted as a *choice-outcome* association, similar to how animals choose a specific lever. However, in contrast to animal versions of this task, a given planet could be presented on the left or right side of the screen, dissociating the choice of a specific reward from a specific motor action.

In the training session, participants received only 1 odor per trial regardless of how many locations they foraged, such that they could build associations between the planets and the types of rewards that could be obtained. In the test phase, they received all outcomes from the foraging phase of one randomly selected trial at the end of each run. This means that they could receive multiple odors if they foraged multiple locations. We have clarified this on page 18 in the description of the test phase:

> Participants were made aware that they would no longer receive odors at the end of each trial. Instead, participants were instructed that at the end of each run, one trial would be randomly selected and all rewards they won on that trial would be delivered (i.e. they could receive multiple deliveries of an odor).

2. The results are dominated by ROI analyses. While sensibly selected ROIs were there any results at the whole brain level. If there were, could these be included in supplementary tables in order to see a more complete picture?

Our *a priori* predictions involved a very limited network of prefrontal and sub-cortical regions that are known to be functionally involved in similar tasks. However, in response to this comment, we conducted an exploratory analysis across all voxels in a gray matter mask of the whole brain, correcting for multiple comparisons using TFCE. This revealed a significant effect of identity-specific reward expectations in the visual cortex (-15, -87, -8, t=6.46, cluster size = 3101, pTFCE<0.001). Follow-up analyses within this functional ROI controlling for visual information showed a null effect (t[29]=0.438, p=0.332), suggesting that this information was likely driven by visual information about the cue. Moreover, we did not find any effects that survived whole-brain TFCE correction for the identity expectation to choice prediction analysis, for the between-participants correlation analyses, or for the information connectivity analysis. We mention this absence of results at the whole-brain level in the revised manuscript.

On page 8:

> An exploratory whole-brain analysis identified an effect in visual cortex ([x,y,z]=[-15, -87, -8], t[29]=6.46 pTFCE<0.001), which was no longer significant when controlling for visual information (t[29]=0.438, p=0.332).

One page 9:

> Further, we did not find any significant decoding at the whole-brain level (pTFCE>0.588)

Page 11:

> Finally, we ran exploratory whole-brain versions of both analyses but did not find any significant clusters (all pTFCE>0.184).

And Page 13:

> No other effects were found in an exploratory whole-brain analysis (pTFCE>0.105).

3. Did the authors consider examining the wider network beyond the amygdala? While there are strong a priori reasons for focusing on the amygdala, my own work in this area (Noonan et al. 2011, JON) also examined the lOFC's functional connectivity

with regions associated with visual form and object representations at the time of informative feedback (i.e. experiencing an error or the first instance of a correct response) and showed that connectivity between the lOFC and perirhinal cortex increased when identify specific information was available to learning. We also saw differences in functional coupling with the lateral habenula/md thalamus. Indeed, my study found that the amygdala showed differential functional connectivity that depended on reward identify at the time of feedback. Given this would be the time to forge the memory this may be a more likely time for the involvement of the amygdala than at the time of choice as explored in the current study. Could the authors consider this wider network and its potential involvement at the time of choice in the context of their own work.

We agree this would be an interesting idea to test. Given studies in rodents have found that outputs from the amygdala to the lOFC drive goal-pursuit in sPIT tasks (e.g., Sais et al. 2018), it could be that amygdala activity in our task supports the mapping of identity expectations to choice. To that end, we created separate regression models for each ROI in which we used activity from the ROI as a moderator on the correlation between the strength of trial-by-trial identity expectations in the lOFC and choices. We focused on the amygdala, perirhinal cortex and mediodorsal thalamus. However, none of these regions showed a significant moderating effect on the relationship between lOFC identity expectations and choice (all $p>0.22$).

Nonetheless, we refer readers to work that has shown links between amygdala activity and learning and decision-making by modifying the sentence on page 14:

> However, prior work in humans focused on linking amygdala BOLD responses to single cue biases[16], consistent with models of amygdala function which propose a role in linking specific reward memories to specific cues during learning[47] and using these memories for decision-making[19,41].

Reviewer #2: Witkowski et al. present an interesting and technically sophisticated fMRI study that attempts to link identity-specific reward representations in lateral orbitofrontal cortex to goal-directed choice behavior via a nucleus accumbens-dorsal anterior cingulate cortex circuit. The core question is important, and the multivariate decoding approach is well-suited to it, and the conclusions are novel. However, there are some methodological issues that somewhat temper the strength of the conclusions. The sample size (n=30) is modest for making claims about multi-region circuitry, particularly between participants, and this should be kept in mind when evaluating effect sizes. In addition, there are concerns about independence of some analyses, causal language, and potentially conflation of identity and value-based choice.

Major

1. The central behavioral claim is that Pavlovian cues bias choices based on the identity of the predicted reward, independent of hedonic value. However, the odor selection procedure can't perfectly match pleasantness within subject (although it does so at the group level), and even small differences in an individual's pleasantness between O1 and O2 could drive apparent identity-specific effects. Fig 2A is quite convincing for behavior in the aligned task condition, but the individual variability does seem to meaningfully relate to differences in pleasantness ratings across individuals, suggesting that this could be a factor throughout the experiment. In the neural analyses, if participants with (e.g.) a stronger O1 preference show both stronger lOFC expectation decoding for O1 and a stronger O1 choice bias, the correlation between lOFC patterns and choice (Figure 4) could partly reflect the neural representation of differential hedonic value rather than identity per se. The cross-decoding control rules out visual cue confounds but does not rule out this value-via-identity confound, and there is no within-design manipulation that orthogonalizes identity from value in the way that, for example, a devaluation procedure would. The authors note that the aligned-condition result is not related to pleasantness differences, but the test here is of a between-participant correlation, which may have limited power with n = 30, so the absence of a significant correlation may not be sufficient to conclude absence of a value contribution. One way to address this might be to include individual-level pleasantness differences scores as a covariate in the regression linking lOFC expectation strength to choice and in the NAc correlation analyses. If the neural effects survive after partialling out value, the identity-based interpretation is much stronger. Similarly, if the lOFC is truly representing reward

identity rather than value, then the multivariate pattern should distinguish O1 from O2 equivalently across participants regardless preference difference, which could be tested. If participants who prefer O2 show stronger lOFC patterns for O2 cues and vice versa, this suggests value

We thank the reviewer for these comments. The dissociation of value and identity is obviously a key component of our study. Because this involves (in some places) interpreting non-significant effect as evidence for the null hypothesis, we computed Bayes Factors to quantify the strength of evidence for the null hypotheses (BF_{01}, i.e., larger values mean stronger evidence for null). We also added the corresponding scatterplots illustrating these non-significant relationships to the supplementary information (Figure S2). We believe the suggested analyses really make the paper stronger.

First, we added text on page 6 to clarify that pleasantness rating differences were not related to choice biases on aligned trials:

> The strength of this overall bias effect was not related to pleasantness or intensity differences between the odors (both p>0.53, both BF_{01}>17.98).

Likewise, the neural effects did not correlate with differences in rated odor pleasantness or intensity (non-cross decoded identity expectations predicting choice x pleasantness difference BF_{01}=23.14, **Fig S2E**; non-cross decoded identity expectations predicting choice x intensity difference BF_{01}=18.88, **Fig S2G**; cross-decoded identity expectations predicting choice x pleasantness difference BF_{01}=8.97 **Fig S2F**), except for cross-decoded identity expectations predicting choice x intensity difference (t[29]=-2.429, p=0.022, BF_{01}=2.14, **Fig S2H**). However, this latter correlation was negative, suggesting that decoding was better when intensity differences were small, indicating that, if anything, intensity differences in this analysis hindered detecting the effect rather than driving it. Because this is difficult to interpret, we report finding no positive relationships on page 9 for brevity, and point readers to the supplementary methods for a full description of the results:

> We did not find any significant positive correlations between any of these decoding results and differences in rated odor pleasantness or intensity (all p's>0.12, all BF_{01}>2.14, see **S2E-H Fig.**).

Secondly, we included pleasantness differences as a covariate for the NAc analyses shown in figures 5A and B. Including pleasantness differences did not change the significant associations between NAc activity and behavioral biases (pTFCE=0.008) and between NAc activity and the lOFC expectation-to-choice decoding (p=0.018). We now report the results of these analyses in the main text on page 10:

> To control for potential confounds related to value, we also included the absolute difference in pleasantness between rewards as a covariate. Paralleling previous work[36,37], we found a significant positive correlation between cue-induced choice bias and activity in NAc at cue onset within an *a priori* ROI of the NAc ([x,y,z]=[-10,6,-8], t[29]=4.18, pTFCE= 0.008; **Fig. 5A**).

On page 10:

> We also included the absolute difference in pleasantness ratings as a covariate in this analysis. We observed a significant effect in the same NAc ROI ([x,y,z]=[-7,8,-10], t[29]=3.62, pTFCE=0.018), showing that participants with greater NAc activation during cue presentation also exhibited greater predictability of choices from lOFC expectation patterns.

And in the methods section on page 23:

> In both cases, we also included the absolute difference between pleasantness ratings for the two odors (collected at the beginning of the second session) as a regressor.

For completeness, we also computed Bayes Factors for the non-significant correlation between pleasantness differences and decoding of identity-specific reward expectations in the aligned condition on page 7:

> These decoding results could not be explained by absolute differences in pleasantness or intensity between the two expected rewards (both pTFCE>0.5, both BF_{01}>21; **S2A and C Fig.**).
>
> This showed significant decoding of identity-specific reward expectations (*t*[29]=2.32, p= 0.014, **Fig. 3C**), which was similarly not driven by differences in pleasantness or intensity ratings (both p>0.25, both BF_{01}>22.00, **S2B and D Fig.**).

Lastly, we tested whether the reward with a higher pleasantness rating for a given person showed stronger decoding compared to the less pleasant reward. We did this for aligned trials by computing separate templates for O1 and O2 and then computed the correlation between trials where O1 or O2 was cued with their respective templates. For competing trials, we computed the correlation for trials where participants chose O1 or O2 and their respective templates. In each case, we then computed the difference in the correlation strength between whichever reward had the higher minus the lower pleasantness rating. In both conditions, independent t-tests were not significant (aligned t[29]=1.195, p=0.121, $B1_{01}$=6.62; competing t[29]=1.490, p=0.074, BF_{01}=4.14, **Fig. S2I**). We also correlated this difference in neural representation with the difference in pleasantness between the odor with the higher and lower pleasantness rating (i.e., the absolute difference between O1 and O2). This correlation was not significant (aligned condition t[29]=-0.342, p=0.735,

BF_{01}=27.46; competing condition t[29]=0.267, p=0.791, BF_{01}=27.35, **Fig. S2J**). Together, these results show that, similar to our previous work (Howard et al. 2015, *PNAS*), the hedonic value of rewards did not affect the decodability of outcome identity in lateral OFC.

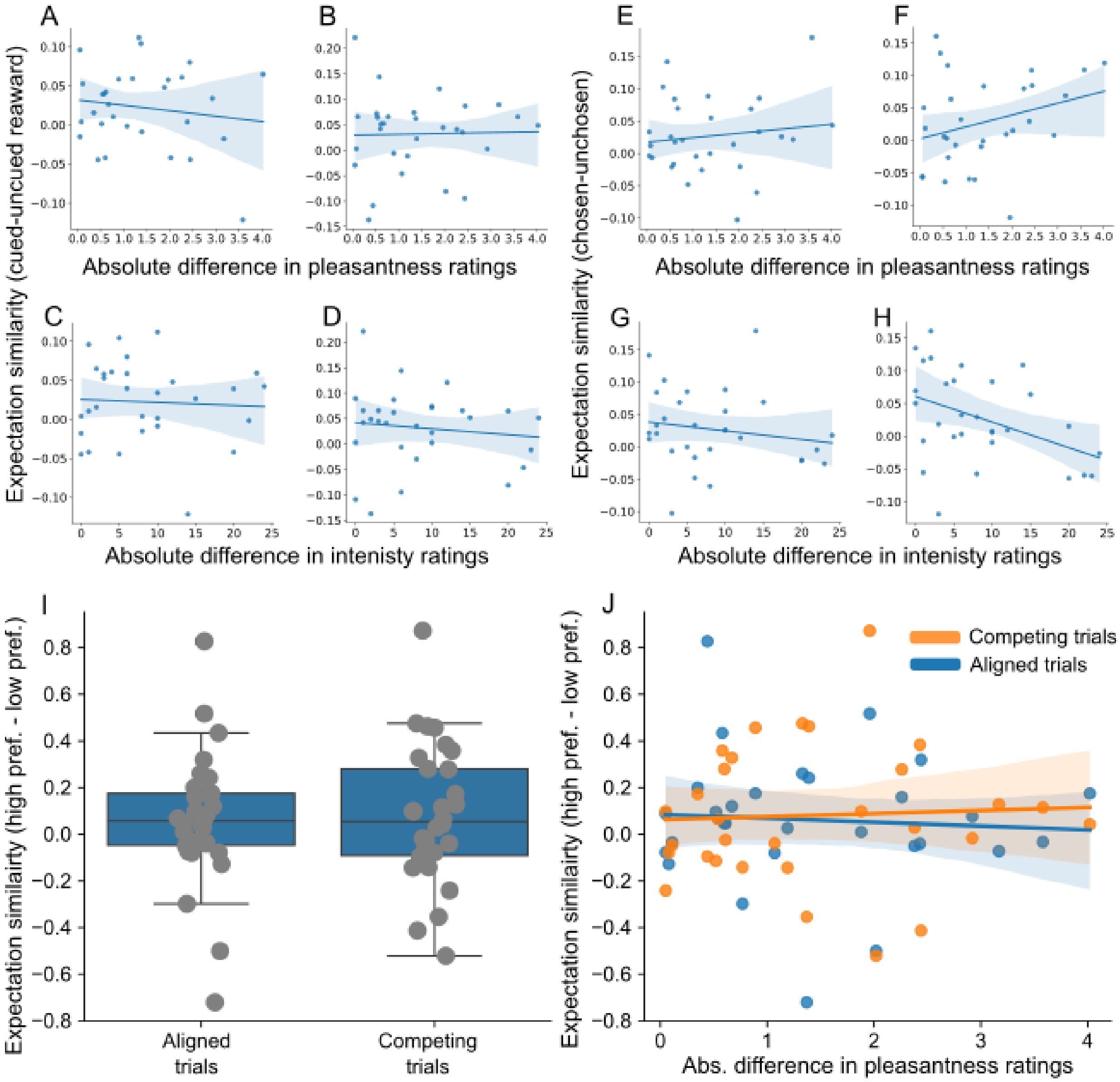

S2 Figure. Control analyses for relationships between perceptual odor ratings and reward identity decoding. A) relationship between decoding of reward identity expectations at the time of the cues in the aligned condition and the absolute difference in pleasantness ratings between the two rewards (t[29]=-0.799, p=0.431, BF_{01}=21.32). B) same as A but for cross-decoding analysis (t[29]=0.136, p=0.893, BF_{01}=29.60) C) relationship between decoding of reward identity expectations at the time of the cues in the aligned condition and the absolute difference in intensity ratings between the two rewards (t[29]=-0.291, p=0.773, BF_{01}=27.17). D) same C but for cross-decoding analysis (t[29]=-0.612, p=0.545, BF_{01}=22.35). E) relationship between correlation between identity expectations and choices in the competing condition and the absolute difference in pleasantness ratings between the two rewards (t[29]=

0.693, p=0.494, BF_{01}=23.14. F) same as E but for cross-decoding analysis (t[29]= 1.619, p=0.117, BF_{01}=8.97). G) relationship between correlation between identity expectations and choices in the competing condition and the absolute difference in intensity ratings between the two rewards (t[29]=-0.903, p=0.374, BF_{01}=18.88). H) same G but for cross-decoding analysis (t[29]=-2.49, p=0.022, BF_{01}=2.41). Results from A-H are summarized in main text. I) For aligned trials we computed separate templates for O1 and O2 and then computed the correlation between trials where O1 or O2 was cued with their respective templates. For competing trials, we computed the correlation between trials where participants chose O1 or O2 and their respective templates. In each case, we then computed the difference in the correlation strength between whichever reward had the higher minus the lower pleasantness rating (i.e. High Pref. – Low Pref.). In both conditions we found null results from independent t-tests (aligned t(29)=1.195, p=0.121, $B1_{01}$=6.62; competing t(29)=1.490, p=0.074, $B1_{01}$=4.14). J) correlation between difference in decoding strength for High Pref minus Low Pref. odors and the absolute difference in pleasantness ratings for each odor (aligned t[29]=-0.342, p=0.735, BF_{01}=27.46; competing t[29]=0.267, p=0.791, BF_{01}=27.35)

2. One of the central neural claims—that lOFC expectation patterns predict subsequent choices—is tested in an ROI defined by voxels that were themselves selected for showing significant expectation decoding in the aligned condition. Although the aligned and competing conditions are different trials, the procedure is still not fully independent: the same voxels are selected for showing strong identity signals and then these are tested for predicting choices. This may inflate the apparent sensitivity of the ROI for the choice-prediction result, and it would be compelling to validate the effect with additional analyses. One approach might be to define ROIs leaving one participant out, so the choice prediction could be tested in participant i with ROIs defined using data from all participants except i. If the choice-prediction result in Figure 4B survives this procedure, it would be much more compelling.

We thank the reviewer for this comment. The reason we generated the lOFC ROI from voxels that showed identity-specific expectations was because we wanted to test if these Pavlovian representations guide choices when pursuing specific types of reward. This, of course, could be true in the aligned condition as well, but because trials in the aligned conditioned only included cues for one type of reward, evoking an expectation and pursuing that reward can be fully yoked. What makes the competing trial distinct is that we present two competing cues and hypothesize that the strength of one expectation over the other determines the choices participant will make. However, this relationship between expectation strength and choice played no role in the definition of our functional ROI. This ROI was selected because it contains identity-specific expectations, and it was then an empirical test (and a main purpose of doing the current study) whether these signals are related to choice.

Of note, as described in our response to point 2.b below, the TFCE-corrected clusters from the searchlight analyses for reward identity decoding on aligned trials overlapped

substantially with TFCE-corrected clusters from the searchlight analyses for identity-expectation to choice decoding on competing trials. Thus, the key finding of overlapping decoding of reward identity and identity-to-choice does not hinge on the selection of a particular ROI within lateral OFC.

However, we agree that it is imperative to test the robustness of this effect with respect to ROI definitions. To that end, we repeated this decoding analysis in a ROI defined using a leave-one-participant-out procedure (threshold is $t(28)=2.467$, $p<.01$). This revealed marginally significant results for the "decoding" and significant results for "cross-decoding" analysis. We have included these results in supplemental figure 3 (**S3 Figure**).

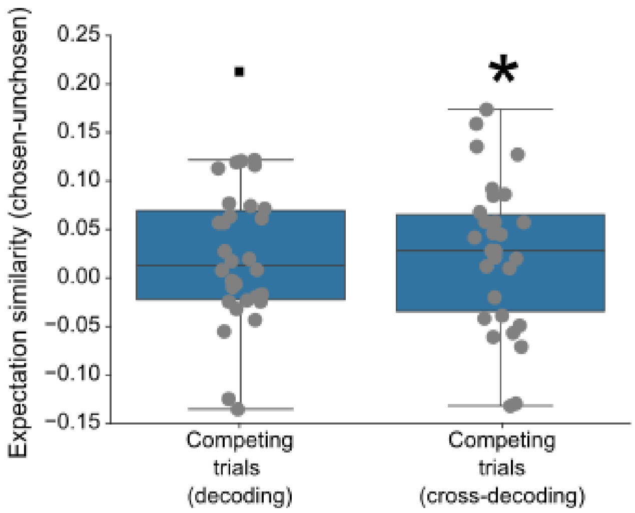

Figure S3. Identity-specific reward expectations in lOFC predict goal-directed choices with alternative ROI definition. A) We compared identity-specific template pattern from the Pavlovian learning task to patterns evoked by competing cues during the test phase of the foraging task, predicting that stronger decoding of one pattern over the other would correspond to subsequent goal-directed choices for that reward at the time of decision. Here, we define the lOFC ROI using a leave-one-out procedure. We started by finding voxels which showed significant decoding of the identity of the expected reward in the aligned condition across all but one participant ($t[28]=2.467$, $p<.01$). We then used this ROI to compute the similarity of trials in which participants chose to pursue a specific reward with that reward's template representation from Pavlovian training trials. We found marginally significant effect when using all Pavlovian training trials in the template ($t[29]=1.656$, $p=0.054$), and a significant effect when we decoded across cue-types to control for visual information ($t[29]=1.841$, $p=0.038$).

a. Two additional analyses would help address the robustness of the primary ROI-based result more directly. The first is reporting the competing-condition choice-prediction result using the full a priori lOFC ROI before any thresholding. This would reveal whether the choice-predictive effect is genuinely specific to voxels that also show aligned-condition decoding, or whether it is more broadly present across the lOFC region (which would have implications for how the cross-condition ROI

procedure should be interpreted). Second, demonstrating that the choice-prediction result in Figure 4B is robust across a range of significance thresholds used to define the aligned-condition ROI would increase confidence in the detected ROI.

To further explore the robustness of our results, we defined ROIs using different thresholds ($p<0.05$ and $p<0.005$). Repeating our decoding analyses in these ROIs, revealed marginally significant decoding ($t[29]= 1.6734$, $p=0.053$) and significant decoding ($t[29]= 2.014$, $p=0.027$) for "decoding" and "cross-decoded", respectively, for the ROI thresholded at $p<0.05$. The ROI thresholded at $p<0.005$ did not reveal any significant effects (both $p>0.184$), perhaps due to the small size of this ROI (15 voxels), but still pointed in the positive direction.

Repeating this analysis in the full anatomical ROI of lateral OFC did not reveal significant effects (both $p>0.313$). This suggests meaningful functional differentiation within the broader lateral OFC, such that effects on choice prediction are confined to subregions that show significant decoding of identity-specific reward expectations (see response to point b below).

b. Relatedly, the aligned and competing conditions differ not only in whether cues agree or conflict, but potentially in the strength and reliability of identity-specific neural representations. In the aligned condition, two cues consistently point to the same reward, which may produce stronger or more coherent lOFC representations than the competing condition, and the assumption that the neural representation of reward identity is stable across the two contexts is non-trivial. It would be interesting to compute the overlap (e.g., Dice coefficient or similar) between voxels showing significant expectation decoding in the aligned condition and voxels where expectation patterns predict choices in the competing condition searchlight. Given the ROI size, they could then test whether spatial overlap is greater than expected by chance.

This is a good point. Figure 4C already shows that there is substantial overlap between the significant clusters from the searchlight analyses for identity-specific reward expectations in aligned trials and expectation-to-choice decoding in the competing condition. This overlap is perhaps the strongest evidence that the same region is involved in both processes since these analyses are agnostic to each other and do not depend on selection of a particular functional ROI. Following the reviewer's suggestion to quantify this overlap, we computed the DICE coefficient between clusters. This revealed a highly significant overlap (Dice = 0.559, $p<.001$, permutation test). We have noted this in the main text on page 9:

> Implementing this analysis in a searchlight revealed a significant cluster in our lOFC ROI ([x,y,z]=[-45, 31, -15], t[29]=3.90, pTFCE=0.015; **Fig.4C**), showing that even a cluster-agnostic analysis showed a highly significant overlap between voxels coding identity-specific expectations and those predicting choice (DICE coefficient = 0.559, p<0.001 permutation test).

3. The causal language throughout the results and discussion, particularly regarding NAc effects, is misplaced. Throughout the text associated with Figures 5 and 6 terms such as "mediates," "gating," "amplifying", and "initiates" all imply a directional mechanistic role that between-participant correlations cannot establish. These results should be presented with more qualifying language. The authors may reasonably note that the analysis in Figure 6 has a built-in temporal structure, but this temporal ordering alone is not sufficient to demonstrate causality. For instance, a common upstream process could, in principle, drive all three signals in sequence without NAc playing a causal modulatory role at all. To the extent the authors wish to support a more mechanistic interpretation, the trial-level time series already extracted for the information connectivity analysis could be submitted to a Granger causality test or a simplified dynamic causal modelling (DCM) analysis to probe whether lOFC fluctuations temporally precede NAc and dACC fluctuations within a trial. These approaches cannot definitively demonstrate causality, but would be a further step toward supporting the interpretations in the text.

This is an important point, and we appreciate the reviewer's careful attention to word choice. It's true that we cannot demonstrate a causal relationship between NAc and the biasing effect, although this interpretation is consistent with previous studies who have shown this causal relationship. We have added qualifying language on page 11 and cited relevant animal work:

> Together, these results suggest that NAc activity mediates the biasing effect of Pavlovian cues on choices, potentially by gating or amplifying the influence of reward-specific expectations in the lOFC on subsequent goal-directed decision making[35,36]

And removed the word "initiates" on page 15:

> Identity-specific reward representations in lOFC are related to biases in goal-pursuit decisions. These biases are moderated by activity in the NAc and flexibly mapped onto reward-obtaining action signals in the dACC.

We also performed the analysis suggested by this reviewer. We used the same regression approach that we used in the main text but compared different temporal orderings of the expectation-to-action information relationship. Specifically, we compared the relationship between the interaction of NAc activity and lOFC expectations representations ($lOFC_E$) at

the cue to dACC action representations ($dACC_A$) at the choice (our original analysis) to three alternative temporal structures: (1) $lOFC_E$ at cue to $dACC_A$ at cue, (2) $lOFC_E$ at choice to $dACC_A$ at choice, and (3) $lOFC_E$ at choice to $dACC_A$ at cue. We then compared the coefficients across orderings, both to zero and to each other. We found that only our original analysis showed a significant effect, and that the coefficient for this ordering was significantly higher than the coefficients for $lOFC_E$ at cue to $dACC_A$ at cue ($t[29]= 1.893$, $p=0.0341$) and $lOFC_E$ at choice to $dACC_A$ at cue ($t[29]=2.119$, $p=0.0214$). We added this analysis to the main text on page 13 and included it as **Fig. 6D**:

Further, the effect in these voxels was specific to the relationship between lOFC expectations at the time of cue and dACC action representations at the time of choice and was not significant for alternative temporal orderings (all ps > 0.08, all $BF_{01} > 38.95$; **Fig. 6D**).

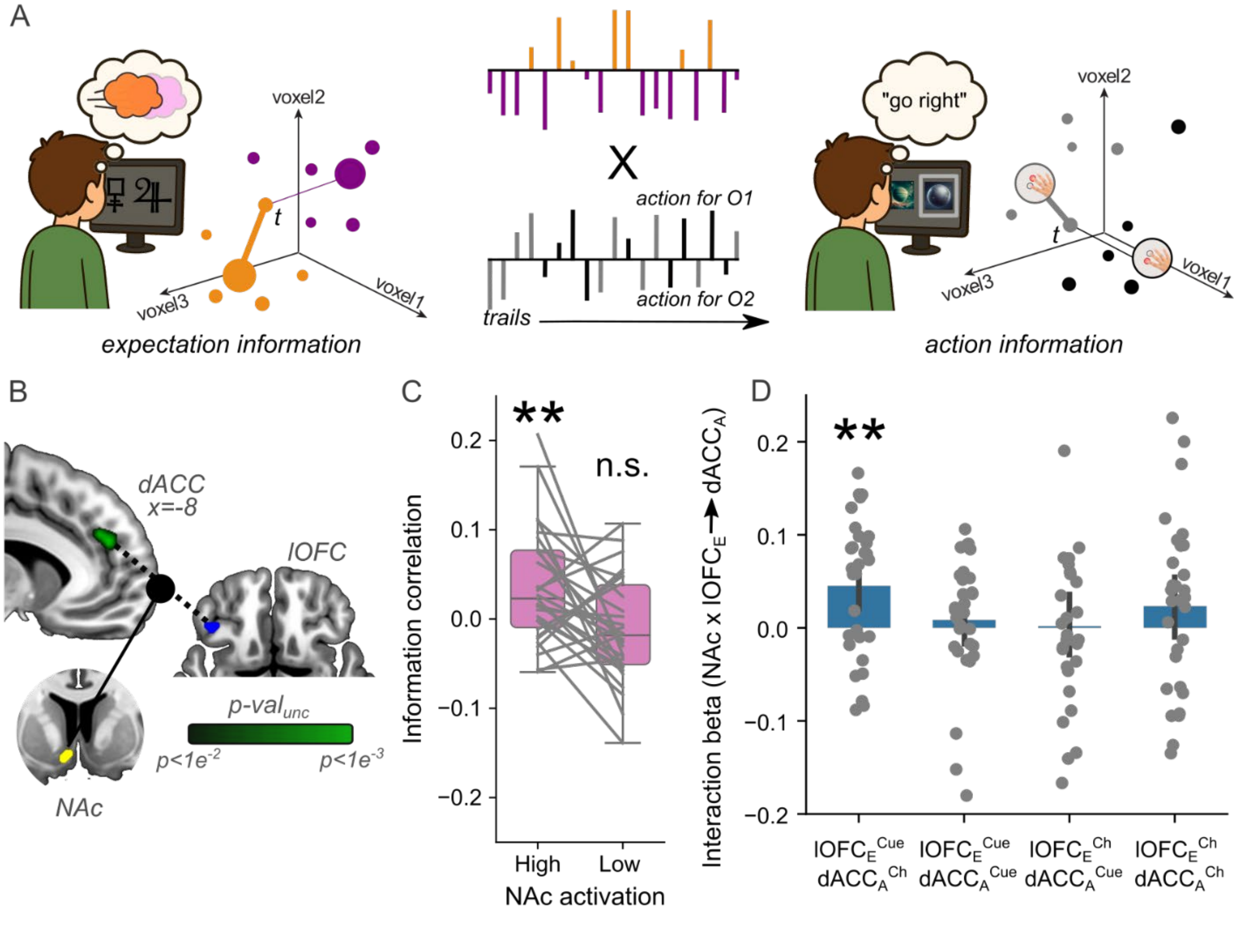

Figure 1. NAc relates reward expectations in lOFC to action representations in dACC. A) *Schematic of the trial-by-trial information measurement of identity-specific reward expectations (left) and action (right). In each trial, we computed the relative distance of neural activity patterns to each of two "expectation" templates. Closer distance to one template (orange line) was taken to be an indicator of stronger expectations for that associated reward. We did the same for action representations at the time of choice (black=left, gray=right) but used a leave-one-out procedure to construct "action" templates by which the representations could be compared. These distances were then projected into the reward identity space such that they reflected the amount of information about the action required to pursue a*

specific reward (see Methods). ***B)*** *Schematic and t-statistic maps showing the results of the information connectivity analysis conducted on the information metrics computed in A. We used a trial-by-trial regression framework to test whether the relationship between lOFC expectation strength and dACC action representations depended on NAc activity, effectively testing NAc's moderating influence on information transfer between lOFC reward expectation and dACC action representations. Maps are displayed using the same conventions as Figure 3.* ***C)*** *Illustration of results shown in C, separated by median-split based on NAc activity, showing that lOFC outcome expectations are positively correlated with dACC action information on trials with high NAc activity.* ***D)*** *Estimated interaction coefficients for alternative temporal orderings of information connectivity. $lOFC_E$ represents the trial-by-trial time course of decoded reward expectations in lOFC and $dACC_A$ represents the time course of decoding actions in dACC. Superscripts indicate the timepoint at which these representations were decoded, "Cue" is at the time of the cue and "Ch" is at the time of choice. ** indicates p<0.01*

4. It seems that the NAc was selected for the analysis relating to lOFC and dACC activity a priori, but I wonder how specific this effect is to this region. If a broader searchlight were used - say for all subcortical structures - would the same pattern emerge elsewhere?

We thank the reviewer for this suggestion. The NAc ROI was selected *a priori* based on previous work in rodents showing its involvement in the relevant behaviors. In response to this comment, we conducted an exploratory analysis using ROIs for each region of the basal ganglia (caudate, putamen, pallidum), substantia nigra pars compacta, amygdala, hippocampus, and medial dorsal thalamus. These ROI's were derived from the same atlas our NAc ROI (the Anatomical Automatic Labeling atlas; Rolls et al, 2020).

We first tested whether voxels in any of these regions were correlated either with choice biases or the extent to which lOFC identity expectations predicted choices. In the first case, we found a significant effect in the left pallidum (pTFCE=0.043), which did not survive correction for multiple comparisons across ROIs (pFDR= 0.3702). No other regions showed a significant effect. In the second case, no regions showed a significant effect.

However, neither of these results preclude the involvement of these regions in the information connectivity analysis. We repeated the searchlight analysis from the main text, using trial-by-trial univariate cue-evoked activity in each ROI to create interaction regressors with lOFC expectation decoding to predict action decoding in dACC. Several ROIs showed nominally significant effects, but only the effect in the nucleus accumbens ROI survived FDR correction. Below are the uncorrected and FDR corrected p-values:

Region	p-value	FDR
Caudate	0.2854	0.2948
Pallidum	0.2948	0.2948
Putamen	0.0468	0.1117
SN	0.0698	0.1117
Amygdala	0.0656	0.1117
MD Thalamus	0.0946	0.1261
Hippocampus	0.0134	0.0536
NAc	0.006	0.048

S1 Table. Alternative moderating ROIs for information connectivity analysis. To test whether other subcortical regions may be involved in information transfer from lOFC to dACC, we repeated the searchlight analysis from the main text using subcortical rois from the AAL (caudate, putamen, pallidum and substantia nigra pars compacta, the amygdala, hippocampus, the medial dorsal thalamus, and the nucleus accumbens). We used the trial-by-trial univariate cue-evoked activity in each ROI as an interaction regressor with lOFC expectation decoding to predict action decoding in dACC. We found that after correction for multiple comparisons, only the NAc ROI showed a significant effect.

We direct readers to these results in the main text on page 13:

> Similar effects were also not significant when using activity in other subcortical areas instead of NAc (see **S1 Table**). These results suggest that NAc activity may amplify the influence of Pavlovian cues on choice by strengthening the link between identity-specific reward expectations in lOFC and action representations in dACC.

Minor

1. The foraging task has several interesting behavioral variables (response times, selection patterns, etc.) that could potentially provide converging evidence for neural results. For instance, do participants with high NAc activation show not only stronger lOFC-to-choice links but also faster or more consistent choices on aligned trials?

This is an interesting suggestion. In response, we conducted a between participants analysis attempting to link NAc activation at the time of the cue to response times at the time of choice. We did not find a significant difference between response times in trials where participants did versus did not follow the cue in aligned trials ($t[29]=-1.426$, $p=0.082$). Nonetheless, we did the analysis considering both the difference in response time between these conditions and the response time on trials where participants followed the cue. We included these in the same model given that they showed only a slight correlation ($r=0.23$). However, we did not find any significant correlation between either of these variables and activation at the time of the cue in NAc (both $pTFCE>0.22$). We also correlated NAc activation with the number of planets foraged by participants and similarly did not find a significant effect ($pTFCE>0.451$). This suggests that the NAc activation in

response to the cue may specifically support the selection of options and not the vigor of responses.

2. The authors could exploit the temporal structure within trials more explicitly. They already use a finite impulse response GLM to show that lOFC expectation representations precede dACC action representations. Similar approaches might be applied to the trial-by-trial information connectivity result. For instance, does the lOFC→dACC information coupling emerge earlier in trials with high NAc activity than in trials with low NAc activity? If so, this would be consistent with NAc enabling faster propagation of identity signals.

This is an interesting idea and would be consistent with findings in non-human primates (Balewski, et al. 2023). However, the pattern of results in figure 6 suggests no substantial coupling between lOFC and dACC at the time of choice in "low NAc" trials. This would seem to preclude finding timing differences between each condition in our data because that signal does not appear to begin with. Further, if it were the case that it's the delay in the coupling that is making the signal noisier/undetectable, it is still unlikely that we could see this in the finite impulse response model. In non-human primates, the time-course of the ACC ramping signal tends to last about 1 second. Our TR is 1.5 seconds, making it very difficult to detect any changes in the ramping signal that are within our response time window. Therefore, we leave this question to future studies with higher temporal precision to investigate.

3. The use of median split in Figure 6C to illustrate the NAc moderation effect should be accompanied by the continuous moderation analysis plotted as an interaction plot, or the authors should at least confirm that the two halves of the median split do not differ on any other relevant variables (odor pleasantness difference, overall choice bias, motion parameters) that could confound the interpretation.

We thank the reviewer for this comment. We chose not to show this one as a continuous interaction plot in the main text for ease of interpretation and clarity with respect to the distribution of effects seen across participants. Making this into a continuous interaction plot involves plotting 62 lines (one line per trial group for each participant, and 2 for the mean of each trial group) and makes it difficult to illustrate the difference in slopes for each participant (which are readily observable in the median split plot). However, we have included the requested figure below for the reviewer to evaluate. On the X-axes are the normalized differences in the distance between the trial-by-trial outcome expectations representation and each template pattern in lOFC. On the Y-axes is the same but for information about chosen actions in dACC. The coefficients for high and low NAc activation trials are plotted in the left and right panels, respectively. The black lines represent the

mean coefficients across participants.

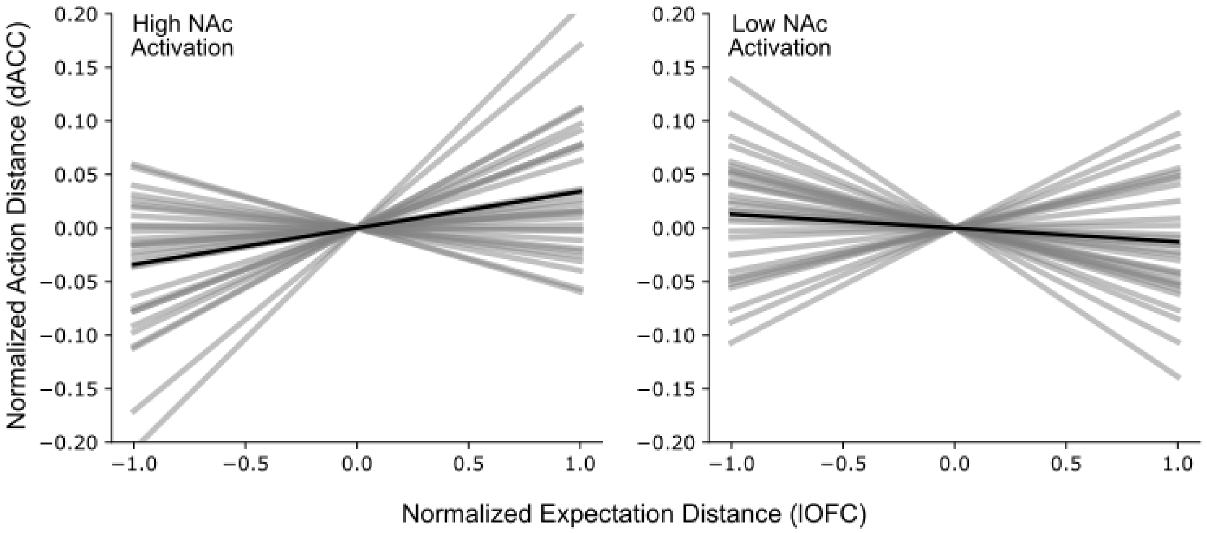

We also conducted several analyses to rule out potential confounds. Most importantly, we did not find any correlation between this effect and the absolute difference in pleasantness ratings between O1 and O2 ($t[29]=0.795$, $p=0.433$, $BF_{01}=21.03$). We also compared a number of measures between trials with high and low NAc activity and did not find any significant differences, specifically the average head motion ($t[29]= 0.224$, $p=0.8247$), the number of each type of reward selected ($t[29]= 0.513$, $p=0.6119$), the number of aligned versus competing trials ($t[29]= -0.4819$, $p=0.6335$), or the average odor pleasantness ($t[29]=-0.4936$, $p=0.625$) .

4. The information connectivity analysis (Figure 6) pools across both aligned and competing trials, and the regression model appropriately includes the identity of the reward pursued on each trial as a separate regressor. In the competing condition, two conflicting identities may make lOFC expectation measures noisier or more ambiguous, potentially attenuating or distorting the lOFC-dACC relationship in ways that are difficult to characterize. The authors should confirm that the information connectivity result holds when the analysis is restricted to aligned trials only, and report whether the effect sizes differ between aligned and competing trial types. If the effect size is comparable, this would strengthen the interpretation of results reported across all trials.

We thank the reviewer for this suggestion because it's important to make sure that one condition is not driving this result. We repeated the analysis considering only aligned or competing trials. We did not find a significant difference between conditions, suggesting that the effect size was comparable across conditions (pTFCE>0.33). Further, neither

condition was significant by itself (aligned condition pTFCE=0.15; competing condition pTFCE=0.33), suggesting that splitting trials into aligned and competing trials left the analysis underpowered. We noted the absence of a difference between conditions in the results on page 13:

> Follow up analysis showed that this effect did not differ between aligned and competing trials (pTFCE>0.33)

5. Figure S2 is central to the manuscript's narrative, and the authors might consider including it in the main text (in Figure 6 or on its own).

We thank the reviewer for this suggestion. The original purpose of this analysis was to confirm what is implied by the analyses described in the main text. That is, to show that identity specific expectations appear before choices even if the GLM is agonistic to the temporal structure of the task. This finding is further supported by the analysis the reviewer suggested (major point 3) and which shows more directly that temporal ordering of the information is critical for this analysis to work. We have included this result as **Fig.6D,** but decided to leave Figure S2 in the supplement (now Fig S4).

6. The Methods mention that "trials where participants did not respond within the response window were repeated at the end of the block" in the learning phase of the foraging task. If this was also true during the test phase as well, descriptive statistics on the frequency of such misses across aligned and competing trials would be informative.

We appreciate this comment. This was not true of the test phase. We have clarified this ambiguity and reported the mean number of missing trials across trial types on page 18:

> Participants did not repeat trials in which they failed to respond during planet selection (missed trials Mean =1.5%, SD=1.6%).

We report the mean across trial types because we did not find any difference between aligned and competing conditions ($t[29]=1.07$, $p=0.293$). We repeated missed trials during training to ensure that exposure to both odors was as matched as possible. However, this was not an issue in the test phase, in which odors were delivered at the end of each run.
