# Supplementary material for: Identity-specific reward expectations in orbitofrontal cortex guide goal-directed choices
Source: PLoS Biol. 2026 Jul 9;24(7):e3003829. doi: 10.1371/journal.pbio.3003829 (PMC13349123; doi:10.1371/journal.pbio.3003829)
Supplement: Attachment — Submitted filename: point-by-point responses_R2.pdf [file pbio.3003829.s009.pdf]

Reviewer #2: The authors have responded thoughtfully to our concerns, and we find the manuscript significantly improved. The addition of Bayes Factor analyses, pleasantness covariates, the DICE coefficient overlap analysis, and the temporal ordering comparison (Figure 6D) all meaningfully strengthen the paper. In particular, the temporal ordering analysis in Figure 6D—showing that the lOFC expectation-to-dACC action relationship is specific to the cue-to-choice window and does not hold for alternative temporal orderings—is a compelling addition that provides the clearest evidence in the paper for a directional flow of information from reward expectations to action selection. Careful attention to potential effects of pleasantness rating differences in the updating analyses also significantly strengthens claims about identity vs. value. Our main outstanding suggestion is that these efforts be better integrated into the manuscript, which we elaborate on below.

The authors made considerable efforts with new analyses to demonstrate that their central claims are robust and cannot be explained by other means, but in places these additional analyses are not well integrated into the revised manuscript's narrative. We appreciate that the authors may not want to include all additional analyses/visualizations in the manuscript, but some of them would make the manuscript clearer and more compelling. Specific suggestions are below:

We are glad to hear we have been able to address the reviewers' comments and that they find the manuscript to be significantly improved. We have addressed the remaining suggestions as outlined below.

a. Exploratory whole brain analyses conducted in response to Reviewer 1's Point 2 failed to find any significant clusters. It would be helpful if this negative result were discussed in further detail, including ways in which it might temper interpretations.

This is an interesting point. We have noted the absence of effects on the whole brain level in the discussion on page 14:

> Note that we did not find representations of identity-specific outcome expectations in an exploratory whole-brain analysis, suggesting that these effects may be restricted to the lOFC.

b. Regression models evaluating moderating effects of amygdala, perirhinal, and MD thalamus activity on the relationship between lOFC identity expectations and

choice found no effect in response to Reviewer 1's Point 3. The lack of an effect here, particularly in amygdala, is likely to be interesting to readers.

We now mention this analysis in the discussion section where we discuss our null findings for the amygdala on page 15:

> This stands in contrast to an exploratory analysis suggested by one of the reviewers, which did not reveal evidence for a moderating effect of amygdala, mediodorsal thalamus or perirhinal cortex on the information connectivity between lOFC and dACC.

c. The follow up analyses using leave-one-participant-out are interesting and informative. The fact that this analysis resulted in only a marginally significant effect should be discussed and acknowledged in the Results. The results in Figure S3 should be referenced in the updated manuscript.

We thank the reviewer for this point and agree that it's important to reference these results in the manuscript. We have now added these results on page 9:

> However, this analysis was somewhat sensitive to ROI definition. Using a leave-one-participant-out cross-validation procedure for defining this ROI showed only a marginally significant effect for decoding (t[29]=1.656, p=0.054), but a significant effect when cross-decoding (t[29]=1.841, p=0.038), indicating that the strength of these effects may depend on the ROI definition (see **S3 Figure**).

d. The analyses carried out in response to Major Point 2a are very informative and merit inclusion in the revised manuscript. The authors note in their response to reviewers that the choice-prediction effect fails to survive when tested in the full anatomical lOFC ROI prior to any functional thresholding, suggesting the effect is genuinely confined to a subset of lOFC voxels that also show identity decoding. The DICE result provides independent support for the co-localization of these signals, but it's still not clear whether the ROI-based choice-prediction result would replicate in an independent sample. It would help if the authors add a short paragraph to the Discussion that directly addresses this issue—acknowledging that the choice-prediction result is sensitive to ROI definition, that it is marginal under the leave-one-out procedure, and that the DICE-based spatial overlap provides the strongest independent evidence for co-localization of expectation and choice-predictive signals in lOFC. This would not undermine the paper's conclusions but would give readers the information they need to contextualize the finding appropriately.

We agree that these results should be discussed more fully because they imply an interesting point – that the sub-region of lOFC required for determining choices in the competing condition is not necessarily coextensive with those signaling the identity-specific expectations. We have discussed this on page 14:

> Importantly, follow-up analyses revealed additional information about the anatomical localization of these effects within lOFC. The cluster of voxels showing the choice-prediction effect survived correction within an anatomical ROI of lOFC but the effect was not significant across the entire ROI, suggesting it is restricted to a subregion within lOFC. This cluster significantly overlapped with voxels coding identity-specific expectations, but the overlap was moderate, and constraining the choice-prediction analysis to voxels that represented identity-specific expectations was somewhat sensitive to the exact selection criteria (**S3 Figure**). Together, this suggests that areas of lOFC coding identity-specific reward expectations when cues predict the same reward may only partially overlap with those recruited to resolve competition when multiple cues signal different reward expectations.
