# Supplementary material for: Identity-specific reward expectations in orbitofrontal cortex guide goal-directed choices
Source: PLoS Biol. 2026 Jul 9;24(7):e3003829. doi: 10.1371/journal.pbio.3003829 (PMC13349123; doi:10.1371/journal.pbio.3003829)
Supplement: Peer review history

# Editor report: Decision Letter 0

**DOI:** 10.1371/journal.pbio.3003829.r001

**Authors:** Christian Schnell, PhD

10 Feb 2026

Dear Thorsten,

Thank you for submitting your manuscript entitled "Identity-specific reward expectations in orbitofrontal cortex guide goal-directed choices" for consideration as a Research Article by PLOS Biology, and apologies for the technical issues during the previous full submission.

Your manuscript has now been evaluated by the PLOS Biology editorial staff as well as by an academic editor with relevant expertise and I am writing to let you know that we would like to send your submission out for external peer review.

However, before we can send your manuscript to reviewers, we need you to complete your submission by providing the metadata that is required for full assessment. To this end, please login to Editorial Manager where you will find the paper in the 'Submissions Needing Revisions' folder on your homepage. Please click 'Revise Submission' from the Action Links and complete all additional questions in the submission questionnaire.

Once your full submission is complete, your paper will undergo a series of checks in preparation for peer review. After your manuscript has passed the checks it will be sent out for review. To provide the metadata for your submission, please Login to Editorial Manager (https://www.editorialmanager.com/pbiology) within two working days, i.e. by Feb 12 2026 11:59PM.

If your manuscript has been previously peer-reviewed at another journal, PLOS Biology is willing to work with those reviews in order to avoid re-starting the process. Submission of the previous reviews is entirely optional and our ability to use them effectively will depend on the willingness of the previous journal to confirm the content of the reports and share the reviewer identities. Please note that we reserve the right to invite additional reviewers if we consider that additional/independent reviewers are needed, although we aim to avoid this as far as possible. In our experience, working with previous reviews does save time.

If you would like us to consider previous reviewer reports, please edit your cover letter to let us know and include the name of the journal where the work was previously considered and the manuscript ID it was given. In addition, please upload a response to the reviews as a 'Prior Peer Review' file type, which should include the reports in full and a point-by-point reply detailing how you have or plan to address the reviewers' concerns.

During the process of completing your manuscript submission, you will be invited to opt-in to posting your pre-review manuscript as a bioRxiv preprint. Visit http://journals.plos.org/plosbiology/s/preprints for full details. If you consent to posting your current manuscript as a preprint, please upload a single Preprint PDF.

Feel free to email us at plosbiology@plos.org if you have any queries relating to your submission.

Kind regards,

Christian

Christian Schnell, PhD

Senior Editor

PLOS Biology

cschnell@plos.org

# Peer review: Decision Letter 1

**DOI:** 10.1371/journal.pbio.3003829.r002

**Authors:** Christian Schnell, PhD

25 Mar 2026

Dear Thorsten,

Thank you for your patience while your manuscript "Identity-specific reward expectations in orbitofrontal cortex guide goal-directed choices" was peer-reviewed at PLOS Biology. It has now been evaluated by the PLOS Biology editors, an Academic Editor with relevant expertise, and by several independent reviewers.

In light of the reviews, which you will find at the end of this email, we would like to invite you to revise the work to thoroughly address the reviewers' reports.

As you will see below, both reviewers overall think that your study is well executed and provides important insights. While Reviewer 1 has a few relatively minor suggestions, Reviewer 2 raises a number of more major concerns that can be addressed with additional analyses to rule out alternative explanations, and by exercising more caution with regards to claims of causality.

Given the extent of revision needed, we cannot make a decision about publication until we have seen the revised manuscript and your response to the reviewers' comments. Your revised manuscript is likely to be sent for further evaluation by all or a subset of the reviewers.

In addition to these revisions, you will need to complete some formatting changes, which you will receive in a follow up email. A member of our team will be in touch with a set of requests shortly.

We expect to receive your revised manuscript within 3 months. Please email us (plosbiology@plos.org) if you have any questions or concerns, or would like to request an extension.

At this stage, your manuscript remains formally under active consideration at our journal; please notify us by email if you do not intend to submit a revision so that we may withdraw it.

**IMPORTANT - SUBMITTING YOUR REVISION**

Your revisions should address the specific points made by each reviewer. Please submit the following files along with your revised manuscript:

1. A 'Response to Reviewers' file - this should detail your responses to the editorial requests, present a point-by-point response to all of the reviewers' comments, and indicate the changes made to the manuscript.

*NOTE: In your point-by-point response to the reviewers, please provide the full context of each review. Do not selectively quote paragraphs or sentences to reply to. The entire set of reviewer comments should be present in full and each specific point should be responded to individually, point by point.

You should also cite any additional relevant literature that has been published since the original submission and mention any additional citations in your response.

2. In addition to a clean copy of the manuscript, please also upload a 'track-changes' version of your manuscript that specifies the edits made. This should be uploaded as a "Revised Article with Changes Highlighted" file type.

*Re-submission Checklist*

When you are ready to resubmit your revised manuscript, please refer to this re-submission checklist: https://plos.io/Biology_Checklist

To submit a revised version of your manuscript, please go to https://www.editorialmanager.com/pbiology/ and log in as an Author. Click the link labelled 'Submissions Needing Revision' where you will find your submission record.

Please make sure to read the following important policies and guidelines while preparing your revision:

*Published Peer Review*

Please note while forming your response, if your article is accepted, you may have the opportunity to make the peer review history publicly available. The record will include editor decision letters (with reviews) and your responses to reviewer comments. If eligible, we will contact you to opt in or out. Please see here for more details:

https://blogs.plos.org/plos/2019/05/plos-journals-now-open-for-published-peer-review/

*PLOS Data Policy*

Please note that as a condition of publication PLOS' data policy (http://journals.plos.org/plosbiology/s/data-availability) requires that you make available all data used to draw the conclusions arrived at in your manuscript. If you have not already done so, you must include any data used in your manuscript either in appropriate repositories, within the body of the manuscript, or as supporting information (N.B. this includes any numerical values that were used to generate graphs, histograms etc.). For an example see here: http://www.plosbiology.org/article/info%3Adoi%2F10.1371%2Fjournal.pbio.1001908#s5

*Blot and Gel Data Policy*

We require the original, uncropped and minimally adjusted images supporting all blot and gel results reported in an article's figures or Supporting Information files. We will require these files before a manuscript can be accepted so please prepare them now, if you have not already uploaded them. Please carefully read our guidelines for how to prepare and upload this data: https://journals.plos.org/plosbiology/s/figures#loc-blot-and-gel-reporting-requirements

*Protocols deposition*

To enhance the reproducibility of your results, we recommend that if applicable you deposit your laboratory protocols in protocols.io, where a protocol can be assigned its own identifier (DOI) such that it can be cited independently in the future. Additionally, PLOS ONE offers an option for publishing peer-reviewed Lab Protocol articles, which describe protocols hosted on protocols.io. Read more information on sharing protocols at https://plos.org/protocols?utm_medium=editorial-email&utm_source=authorletters&utm_campaign=protocols

Thank you again for your submission to our journal. We hope that our editorial process has been constructive thus far, and we welcome your feedback at any time. Please don't hesitate to contact us if you have any questions or comments.

Sincerely,

Christian

Christian Schnell, PhD

Senior Editor

PLOS Biology

cschnell@plos.org

------------------------------------

REVIEWS:

Reviewer #1 (MaryAnn Noonan signed her report): Witkowski and colleagues present a paper that examines how reward identity guides choice. They identify a network of regions that transfer reward identity into actions. Central to that is process is the lOFC which codes identify-specific expectations that are related to the subsequent choice. Subsequent analyses suggest these signals are amplified by the nucleus accumbens and potentially translated into action representations in the dACC.

Overall, I think this was an excellent study. The study aims are clear; the task was well designed and the data analysed appropriately. I therefore have limited comments and suggestions.

1. The description of the foraging task was a little unclear to me. In particular why did participants had to forage for the different odours? Did the participants get just a single odour regardless of how many locations they foraged? In standard PIT tasks this would be the part of the task in which the action-outcome associations are formed but here it seems like the action is not important but instead subjects learn another stimulus-outcome association. Is this how the reader should interpret this component of the task?

2. The results are dominated by ROI analyses. While sensibly selected ROIs were there any results at the whole brain level. If there were, could these be included in supplementary tables in order to see a more complete picture?

3. Did the authors consider examining the wider network beyond the amygdala? While there are strong a priori reasons for focusing on the amygdala, my own work in this area (Noonan et al. 2011, JON) also examined the lOFC's functional connectivity with regions associated with visual form and object representations at the time of informative feedback (ie experiencing an error or the first instance of a correct response) and showed that connectivity between the lOFC and perirhinal cortex increased when identify specific information was available to learning. We also saw differences in functional coupling with the lateral habenula/md thalamus. Indeed, my study found that the amygdala showed differential functional connectivity that depended on reward identify at the time of feedback. Given this would be the time to forge the memory this may be a more likely time for the involvement of the amygdala than at the time of choice as explored in the current study. Could the authors consider this wider network and its potential involvement at the time of choice in the context of their own work.

Reviewer #2: Witkowski et al. present an interesting and technically sophisticated fMRI study that attempts to link identity-specific reward representations in lateral orbitofrontal cortex to goal-directed choice behavior via a nucleus accumbens-dorsal anterior cingulate cortex circuit. The core question is important, and the multivariate decoding approach is well-suited to it, and the conclusions are novel. However, there are some methodological issues that somewhat temper the strength of the conclusions. The sample size (n=30) is modest for making claims about multi-region circuitry, particularly between participants, and this should be kept in mind when evaluating effect sizes. In addition, there are concerns about independence of some analyses, causal language, and potentially conflation of identity and value-based choice.

Major

1. The central behavioral claim is that Pavlovian cues bias choices based on the identity of the predicted reward, independent of hedonic value. However, the odor selection procedure can't perfectly match pleasantness within subject (although it does so at the group level), and even small differences in an individual's pleasantness between O1 and O2 could drive apparent identity-specific effects. Fig 2A is quite convincing for behavior in the aligned task condition, but the individual variability does seem to meaningfully relate to differences in pleasantness ratings across individuals, suggesting that this could be a factor throughout the experiment. In the neural analyses, if participants with (e.g.) a stronger O1 preference show both stronger lOFC expectation decoding for O1 and a stronger O1 choice bias, the correlation between lOFC patterns and choice (Figure 4) could partly reflect the neural representation of differential hedonic value rather than identity per se. The cross-decoding control rules out visual cue confounds but does not rule out this value-via-identity confound, and there is no within-design manipulation that orthogonalizes identity from value in the way that, for example, a devaluation procedure would. The authors note that the aligned-condition result is not related to pleasantness differences, but the test here is of a between-participant correlation, which may have limited power with n = 30, so the absence of a significant correlation may not be sufficient to conclude absence of a value contribution. One way to address this might be to include individual-level pleasantness differences scores as a covariate in the regression linking lOFC expectation strength to choice and in the NAc correlation analyses. If the neural effects survive after partialling out value, the identity-based interpretation is much stronger. Similarly, if the lOFC is truly representing reward identity rather than value, then the multivariate pattern should distinguish O1 from O2 equivalently across participants regardless preference difference, which could be tested. If participants who prefer O2 show stronger lOFC patterns for O2 cues and vice versa, this suggests value contamination.

2. One of the central neural claims—that lOFC expectation patterns predict subsequent choices—is tested in an ROI defined by voxels that were themselves selected for showing significant expectation decoding in the aligned condition. Although the aligned and competing conditions are different trials, the procedure is still not fully independent: the same voxels are selected for showing strong identity signals and then these are tested for predicting choices. This may inflate the apparent sensitivity of the ROI for the choice-prediction result, and it would be compelling to validate the effect with additional analyses. One approach might be to define ROIs leaving one participant out, so the choice prediction could be tested in participant i with ROIs defined using data from all participants except i. If the choice-prediction result in Figure 4B survives this procedure, it would be much more compelling.

a. Two additional analyses would help address the robustness of the primary ROI-based result more directly. The first is reporting the competing-condition choice-prediction result using the full a priori lOFC ROI before any thresholding. This would reveal whether the choice-predictive effect is genuinely specific to voxels that also show aligned-condition decoding, or whether it is more broadly present across the lOFC region (which would have implications for how the cross-condition ROI procedure should be interpreted). Second, demonstrating that the choice-prediction result in Figure 4B is robust across a range of significance thresholds used to define the aligned-condition ROI would increase confidence in the detected ROI.

b. Relatedly, the aligned and competing conditions differ not only in whether cues agree or conflict, but potentially in the strength and reliability of identity-specific neural representations. In the aligned condition, two cues consistently point to the same reward, which may produce stronger or more coherent lOFC representations than the competing condition, and the assumption that the neural representation of reward identity is stable across the two contexts is non-trivial. It would be interesting to compute the overlap (e.g., Dice coefficient or similar) between voxels showing significant expectation decoding in the aligned condition and voxels where expectation patterns predict choices in the competing condition searchlight. Given the ROI size, they could then test whether spatial overlap is greater than expected by chance.

3. The causal language throughout the results and discussion, particularly regarding NAc effects, is misplaced. Throughout the text associated with Figures 5 and 6 terms such as "mediates," "gating," "amplifying", and "initiates" all imply a directional mechanistic role that between-participant correlations cannot establish. These results should be presented with more qualifying language. The authors may reasonably note that the analysis in Figure 6 has a built-in temporal structure, but this temporal ordering alone is not sufficient to demonstrate causality. For instance, a common upstream process could, in principle, drive all three signals in sequence without NAc playing a causal modulatory role at all. To the extent the authors wish to support a more mechanistic interpretation, the trial-level time series already extracted for the information connectivity analysis could be submitted to a Granger causality test or a simplified dynamic causal modelling (DCM) analysis to probe whether lOFC fluctuations temporally precede NAc and dACC fluctuations within a trial. These approaches cannot definitively demonstrate causality, but would be a further step toward supporting the interpretations in the text.

4. It seems that the NAc was selected for the analysis relating to lOFC and dACC activity a priori, but I wonder how specific this effect is to this region. If a broader searchlight were used - say for all subcortical structures - would the same pattern emerge elsewhere?

Minor

1. The foraging task has several interesting behavioral variables (response times, selection patterns, etc.) that could potentially provide converging evidence for neural results. For instance, do participants with high NAc activation show not only stronger lOFC-to-choice links but also faster or more consistent choices on aligned trials?

2. The authors could exploit the temporal structure within trials more explicitly. They already use a finite impulse response GLM to show that lOFC expectation representations precede dACC action representations. Similar approaches might be applied to the trial-by-trial information connectivity result. For instance, does the lOFC→dACC information coupling emerge earlier in trials with high NAc activity than in trials with low NAc activity? If so, this would be consistent with NAc enabling faster propagation of identity signals.

3. The use of median split in Figure 6C to illustrate the NAc moderation effect should be accompanied by the continuous moderation analysis plotted as an interaction plot, or the authors should at least confirm that the two halves of the median split do not differ on any other relevant variables (odor pleasantness difference, overall choice bias, motion parameters) that could confound the interpretation.

4. The information connectivity analysis (Figure 6) pools across both aligned and competing trials, and the regression model appropriately includes the identity of the reward pursued on each trial as a separate regressor. In the competing condition, two conflicting identities may make lOFC expectation measures noisier or more ambiguous, potentially attenuating or distorting the lOFC-dACC relationship in ways that are difficult to characterize. The authors should confirm that the information connectivity result holds when the analysis is restricted to aligned trials only, and report whether the effect sizes differ between aligned and competing trial types. If the effect size is comparable, this would strengthen the interpretation of results reported across all trials.

5. Figure S2 is central to the manuscript's narrative and the authors might consider including it in the main text (in Figure 6 or on its own).

6. The Methods mention that "trials where participants did not respond within the response window were repeated at the end of the block" in the learning phase of the foraging task. If this was also true during the test phase as well, descriptive statistics on the frequency of such misses across aligned and competing trials would be informative.

# Author comment: Author response to Decision Letter 2

**DOI:** 10.1371/journal.pbio.3003829.r003

15 Apr 2026

Attachment Submitted filename: point-by-point responses_R1.pdf

# Peer review: Decision Letter 2

**DOI:** 10.1371/journal.pbio.3003829.r004

**Authors:** Christian Schnell, PhD

8 May 2026

Dear Thorsten,

Thank you for your patience while we considered your revised manuscript "Identity-specific reward expectations in orbitofrontal cortex guide goal-directed choices" for publication as a Research Article at PLOS Biology. This revised version of your manuscript has been evaluated by the PLOS Biology editors, the Academic Editor and one the original reviewers.

Based on the reviews and on our Academic Editor's assessment of your revision, we are likely to accept this manuscript for publication, provided you satisfactorily address the remaining points raised by the reviewer and the following data and other policy-related requests:

* Please add the links to the funding agencies in the Financial Disclosure statement in the manuscript details.

* Please mention in the competing interests statement that Thorsten Kahnt is a member of PLOS Biology's editorial board.

* Please include information in the Methods section whether the study has been conducted according to the principles expressed in the Declaration of Helsinki.

* DATA POLICY:

You may be aware of the PLOS Data Policy, which requires that all data be made available without restriction: http://journals.plos.org/plosbiology/s/data-availability. For more information, please also see this editorial: http://dx.doi.org/10.1371/journal.pbio.1001797

Note that we do not require all raw data. Rather, we ask that all individual quantitative observations that underlie the data summarized in the figures and results of your paper be made available in one of the following forms:

1) Supplementary files (e.g., excel). Please ensure that all data files are uploaded as 'Supporting Information' and are invariably referred to (in the manuscript, figure legends, and the Description field when uploading your files) using the following format verbatim: S1 Data, S2 Data, etc. Multiple panels of a single or even several figures can be included as multiple sheets in one excel file that is saved using exactly the following convention: S1_Data.xlsx (using an underscore).

2) Deposition in a publicly available repository. Please also provide the accession code or a reviewer link so that we may view your data before publication.

Regardless of the method selected, please ensure that you provide the individual numerical values that underlie the summary data displayed in the following figure panels as they are essential for readers to assess your analysis and to reproduce it: 1CD, 2AB, 3C, 4B, 6CD, S2I and S3.

NOTE: the numerical data provided should include all replicates AND the way in which the plotted mean and errors were derived (it should not present only the mean/average values).

Please also ensure that figure legends in your manuscript include information on where the underlying data can be found, and ensure your supplemental data file/s has a legend.

Please ensure that your Data Statement in the submission system accurately describes where your data can be found.

* CODE POLICY

Per journal policy, if you have generated any custom code during the course of this investigation, please make it available without restrictions. Please ensure that the code is sufficiently well documented and reusable, and that your Data Statement in the Editorial Manager submission system accurately describes where your code can be found. More information on our Code Policy, what and how to share can be found here: https://journals.plos.org/plosbiology/s/code-availability

Please note that we cannot accept sole deposition of code in GitHub, as this could be changed after publication. However, you can archive this version of your publicly available GitHub code to Zenodo. Once you do this, it will generate a DOI number, which you will need to provide in the Data Accessibility Statement (you are welcome to also provide the GitHub access information). See the process for doing this here: https://docs.github.com/en/repositories/archiving-a-github-repository/referencing-and-citing-content

As you address these items, please take this last chance to review your reference list to ensure that it is complete and correct. If you have cited papers that have been retracted, please include the rationale for doing so in the manuscript text, or remove these references and replace them with relevant current references. Any changes to the reference list should be mentioned in the cover letter that accompanies your revised manuscript.

In addition to these revisions, you may need to complete some formatting changes, which you will receive in a follow up email. A member of our team will be in touch with a set of requests shortly. If you do not receive a separate email within a few days, please assume that checks have been completed, and no additional changes are required.

We expect to receive your revised manuscript within two weeks.

To submit your revision, please go to https://www.editorialmanager.com/pbiology/ and log in as an Author. Click the link labelled 'Submissions Needing Revision' to find your submission record. Your revised submission must include the following:

- a cover letter that should detail your responses to any editorial requests, if applicable, and whether changes have been made to the reference list

- a Response to Reviewers file that provides a detailed response to the reviewers' comments (if applicable, if not applicable please do not delete your existing 'Response to Reviewers' file.)

- a track-changes file indicating any changes that you have made to the manuscript.

NOTE: If Supporting Information files are included with your article, note that these are not copyedited and will be published as they are submitted. Please ensure that these files are legible and of high quality (at least 300 dpi) in an easily accessible file format. For this reason, please be aware that any references listed in an SI file will not be indexed. For more information, see our Supporting Information guidelines:

https://journals.plos.org/plosbiology/s/supporting-information

*Published Peer Review History*

Please note that you may have the opportunity to make the peer review history publicly available. The record will include editor decision letters (with reviews) and your responses to reviewer comments. If eligible, we will contact you to opt in or out. Please see here for more details:

https://plos.org/published-peer-review-history/

*Press*

Should you, your institution's press office or the journal office choose to press release your paper, please ensure you have opted out of Early Article Posting on the submission form. We ask that you notify us as soon as possible if you or your institution is planning to press release the article.

*Protocols deposition*

To enhance the reproducibility of your results, we recommend that if applicable you deposit your laboratory protocols in protocols.io, where a protocol can be assigned its own identifier (DOI) such that it can be cited independently in the future. Additionally, PLOS ONE offers an option for publishing peer-reviewed Lab Protocol articles, which describe protocols hosted on protocols.io. Read more information on sharing protocols at https://plos.org/protocols?utm_medium=editorial-email&utm_source=authorletters&utm_campaign=protocols

Please do not hesitate to contact me should you have any questions.

Sincerely,

Christian

Christian Schnell, PhD

Senior Editor

cschnell@plos.org

PLOS Biology

------------------------------------------------------------------------

Reviewer remarks:

Reviewer #2: The authors have responded thoughtfully to our concerns, and we find the manuscript significantly improved. The addition of Bayes Factor analyses, pleasantness covariates, the DICE coefficient overlap analysis, and the temporal ordering comparison (Figure 6D) all meaningfully strengthen the paper. In particular, the temporal ordering analysis in Figure 6D—showing that the lOFC expectation-to-dACC action relationship is specific to the cue-to-choice window and does not hold for alternative temporal orderings—is a compelling addition that provides the clearest evidence in the paper for a directional flow of information from reward expectations to action selection. Careful attention to potential effects of pleasantness rating differences in the updating analyses also significantly strengthens claims about identity vs. value. Our main outstanding suggestion is that these efforts be better integrated into the manuscript, which we elaborate on below.

The authors made considerable efforts with new analyses to demonstrate that their central claims are robust and cannot be explained by other means, but in places these additional analyses are not well integrated into the revised manuscript's narrative. We appreciate that the authors may not want to include all additional analyses/visualizations in the manuscript, but some of them would make the manuscript clearer and more compelling. Specific suggestions are below:

a. Exploratory whole brain analyses conducted in response to Reviewer 1's Point 2 failed to find any significant clusters. It would be helpful if this negative result were discussed in further detail, including ways in which it might temper interpretations.

b. Regression models evaluating moderating effects of amygdala, perirhinal, and MD thalamus activity on the relationship between lOFC identity expectations and choice found no effect in response to Reviewer 1's Point 3. The lack of an effect here, particularly in amygdala, is likely to be interesting to readers.

c. The follow up analyses using leave-one-participant-out are interesting and informative. The fact that this analysis resulted in only a marginally significant effect should be discussed and acknowledged in the Results. The results in Figure S3 should be referenced in the updated manuscript.

d. The analyses carried out in response to Major Point 2a are very informative and merit inclusion in the revised manuscript. The authors note in their response to reviewers that the choice-prediction effect fails to survive when tested in the full anatomical lOFC ROI prior to any functional thresholding, suggesting the effect is genuinely confined to a subset of lOFC voxels that also show identity decoding. The DICE result provides independent support for the co-localization of these signals, but it's still not clear whether the ROI-based choice-prediction result would replicate in an independent sample. It would help if the authors add a short paragraph to the Discussion that directly addresses this issue—acknowledging that the choice-prediction result is sensitive to ROI definition, that it is marginal under the leave-one-out procedure, and that the DICE-based spatial overlap provides the strongest independent evidence for co-localization of expectation and choice-predictive signals in lOFC. This would not undermine the paper's conclusions but would give readers the information they need to contextualize the finding appropriately.

# Author comment: Author response to Decision Letter 3

**DOI:** 10.1371/journal.pbio.3003829.r005

13 May 2026

Attachment Submitted filename: point-by-point responses_R2.pdf

# Editor report: Decision Letter 3

**DOI:** 10.1371/journal.pbio.3003829.r006

**Authors:** Christian Schnell, PhD

15 May 2026

Dear Thorsten,

Thank you for the submission of your revised Research Article "Identity-specific reward expectations in orbitofrontal cortex guide goal-directed choices" for publication in PLOS Biology. On behalf of my colleagues and the Academic Editor, Raphael Kaplan, I am pleased to say that we can in principle accept your manuscript for publication, provided you address any remaining formatting and reporting issues. These will be detailed in an email you should receive within 2-3 business days from our colleagues in the journal operations team; no action is required from you until then. Please note that we will not be able to formally accept your manuscript and schedule it for publication until you have completed any requested changes.

When you attend to these requests, please also ensure that all corresponding figure legends in your manuscript include information on where the underlying data can be found, for example: Source data can be found at 'doi.org/10.17605/OSF.IO/24DKW'".

Please take a minute to log into Editorial Manager at http://www.editorialmanager.com/pbiology/, click the "Update My Information" link at the top of the page, and update your user information to ensure an efficient production process.

PRESS

We frequently collaborate with press offices. If your institution or institutions have a press office, please notify them about your upcoming paper at this point, to enable them to help maximise its impact. If the press office is planning to promote your findings, we would be grateful if they could coordinate with biologypress@plos.org. If you have previously opted in to the early version process, we ask that you notify us immediately of any press plans so that we may opt out on your behalf.

We also ask that you take this opportunity to read our Embargo Policy regarding the discussion, promotion and media coverage of work that is yet to be published by PLOS. As your manuscript is not yet published, it is bound by the conditions of our Embargo Policy. Please be aware that this policy is in place both to ensure that any press coverage of your article is fully substantiated and to provide a direct link between such coverage and the published work. For full details of our Embargo Policy, please visit http://www.plos.org/about/media-inquiries/embargo-policy/.

Thank you again for choosing PLOS Biology for publication and supporting Open Access publishing. We look forward to publishing your study.

Sincerely,

Christian

Christian Schnell, PhD

Senior Editor

PLOS Biology

cschnell@plos.org
